# Evolution of the myosin heavy chain gene *MYH14* and its intronic microRNA miR-499: muscle-specific miR-499 expression persists in the absence of the ancestral host gene

**DOI:** 10.1186/1471-2148-13-142

**Published:** 2013-07-06

**Authors:** Sharmin Siddique Bhuiyan, Shigeharu Kinoshita, Chaninya Wongwarangkana, Md Asaduzzaman, Shuichi Asakawa, Shugo Watabe

**Affiliations:** 1 Department of Aquatic Bioscience, Graduate School of Agricultural and Life Sciences, The University of Tokyo, Bunkyo, Tokyo 113-8657, Japan; 2 School of Marine Bioscience, Kitasato University, Minami, Sagamihara, Kanagawa 252-0373, Japan

**Keywords:** Myosin heavy chain, *MYH14* (*MYH7b*), microRNA, miR-499, Muscle, Muscle fiber-type, Teleostei

## Abstract

**Background:**

A novel sarcomeric myosin heavy chain gene, *MYH14*, was identified following the completion of the human genome project. *MYH14* contains an intronic microRNA, miR-499, which is expressed in a slow/cardiac muscle specific manner along with its host gene; it plays a key role in muscle fiber-type specification in mammals. Interestingly, teleost fish genomes contain multiple *MYH14* and miR-499 paralogs. However, the evolutionary history of *MYH14* and miR-499 has not been studied in detail. In the present study, we identified *MYH14*/miR-499 loci on various teleost fish genomes and examined their evolutionary history by sequence and expression analyses.

**Results:**

Synteny and phylogenetic analyses depict the evolutionary history of *MYH14*/miR-499 loci where teleost specific duplication and several subsequent rounds of species-specific gene loss events took place. Interestingly, miR-499 was not located in the *MYH14* introns of certain teleost fish. An *MYH14* paralog, lacking miR-499, exhibited an accelerated rate of evolution compared with those containing miR-499, suggesting a putative functional relationship between *MYH14* and miR-499. In medaka, *Oryzias latipes*, miR-499 is present where *MYH14* is completely absent in the genome. Furthermore, by using *in situ* hybridization and small RNA sequencing, miR-499 was expressed in the notochord at the medaka embryonic stage and slow/cardiac muscle at the larval and adult stages. Comparing the flanking sequences of *MYH14*/miR-499 loci between torafugu *Takifugu rubripes*, zebrafish *Danio rerio*, and medaka revealed some highly conserved regions, suggesting that *cis*-regulatory elements have been functionally conserved in medaka miR-499 despite the loss of its host gene.

**Conclusions:**

This study reveals the evolutionary history of the *MYH14*/miRNA-499 locus in teleost fish, indicating divergent distribution and expression of *MYH14* and miR-499 genes in different teleost fish lineages. We also found that medaka miR-499 was even expressed in the absence of its host gene. To our knowledge, this is the first report that shows the conversion of intronic into non-intronic miRNA during the evolution of a teleost fish lineage.

## Background

To meet the constantly changing functional demands, the physiological properties of skeletal muscle are highly adjustable and are achieved through a process of switching muscle fiber-types, such as slow and fast muscle fibers, in response to internal and external stimuli, a process termed muscle fiber-type plasticity [1]. Myosin heavy chains (MYHs) form a large gene family that includes sarcomeric MYHs, major contractile proteins of striated muscles that are expressed in a spatio-temporal manner defining the functional properties of different muscle fiber subtypes [1]. In humans, sarcomeric *MYHs* form two clusters on the genome where skeletal and cardiac *MYHs* are arrayed in tandem on chromosome Chr17 and Chr14, respectively [2-5]. Upon completion of the human genome project, a novel *MYH* named *MYH14* (*MYH7b*) was identified on Chr20 [6], recently, there has been increasing interest in its direct involvement in muscle fiber-type plasticity. Mammalian *MYH14* has a microRNA, miR-499, in its 19th intron that suppresses the expression of genes involved in muscle fiber-type specification [7-11]; thus, miR-499 seemingly acts to support normal slow-muscle formation in mammals.

Our previous studies revealed that teleost fish also have *MYH14* in their genomes [12,13]. Expression analysis in torafugu *Takifugu rubripes* Abe 1949 and zebrafish *Danio rerio* Hamilton 1822 revealed that *MYH14* is one of the major components of the *MYH* repertoire expressed in the slow and cardiac muscles of teleost fish [14,15], suggesting its role in teleost muscle formation. Consistent with functional conservation with mammals, Wang et al. [16] showed that the transcriptional network of Sox6/*MYH14*/miR-499 plays an essential role in maintaining slow muscle lineage in larval zebrafish muscle. Our previous study also showed that teleost fish contain a higher number of *MYHs* in their genomes than do their mammalian counterparts [12,13,17,18]. Two *MYH14* paralogs, *MYH*_*M3383*_ and *MYH*_*M5*_, were identified in the torafugu genome by phylogenetic and syntenic analyses [13]. Moreover, we have also previously found that medaka *Oryzias latipes* lacks *MYH14* in the syntenic region [15]. These lines of evidence allowed us to speculate on the existence of a highly varied distribution and function of *MYH14* and miR-499 in teleost fish.

The aim of this study was to elucidate the evolutionary history of *MYH14*/miR-499 in fish. *MYH14* and miR-499 genes were screened from available vertebrate genome databases, and their evolutionary history was examined by synteny and phylogenetic analyses. In this study, we confirm the conversion of intronic into intergenic miRNA during fish evolution.

## Results

### Distribution of MYH14 and miR-499 in teleost fish genomes

**Figure 1:**
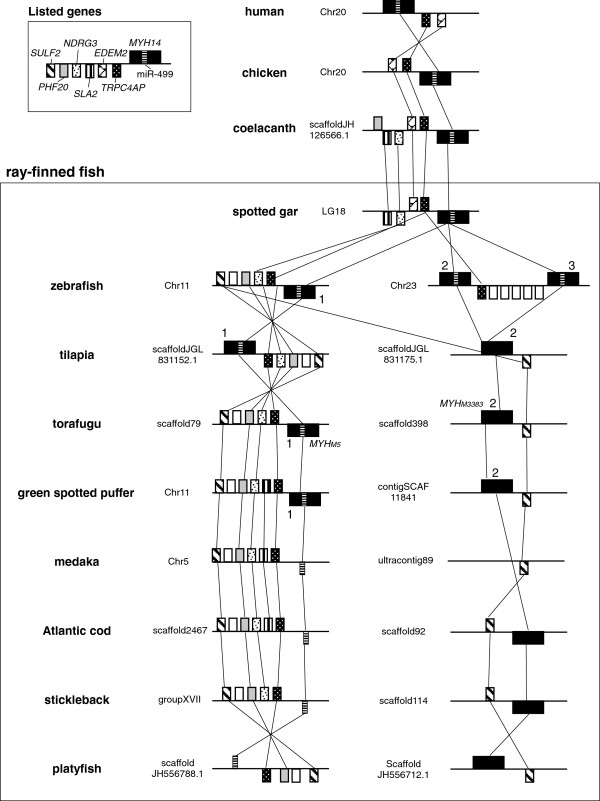
**Genomic organization of *MYH14 *and miR-499 in various vertebrates.** Orthologous genes are connected by solid and dotted lines. Genes displayed above the midline are in forward strands (+ orientation, from left to right), whereas those displayed below are in reverse strands (− orientation, from right to left). *MYH14* and miR-499 paralogs found in one species are distinguished by numbers (see Table 1). Abbreviations used: Chr, chromosome; TRPC4AP, transient receptor potential cation channel, subfamily C, member 4 associated protein; EDEM2, ER degradation enhancer, mannosidase alpha-like 2; SLA2, Src-like-adaptor 2; NDRG3, N-myc downstream regulated family member 3; PHF20, PHD finger protein 20; *SULF2*, sulfatase 2.

Using the genomic databases available for different vertebrates, we examined the syntenic organization of human *MYH14* and miR-499 with their orthologs. The locations and IDs of *MYH14* and miR-499 used in this study are shown in Table 1 and Figure 1. Our results show that the tandem arrayed location of the ER degradation enhancer, mannosidase alpha-like 2 gene (*EDEM2*), transient receptor potential cation channel subfamily C member 4 associated protein gene (*TRPC4AP*), and *MYH14* containing miR-499 were conserved in humans, chickens, and coelacanths *Latimeria chalumnae*. The synteny was also found LG18 in spotted gar *Lepisosteus oculatus*. In zebrafish Chr11, *MYH14* containing miR-499 was located next to *TRPC4AP*. In addition, two *MYH14s* were also found on Chr23 located near a putative *TRPC4AP* paralog. Both zebrafish *MYH14* contained miR-499, totaling three *MYH14*/miR-499 pairs in this species. Ikeda et al. [13] reported two *MYH14* paralogs, *MYH*_*M5*_ and *MYH*_*M3383*_, in the torafugu genome. The former was located on scaffold79 and the latter on scaffold398. *MYH*_*M5*_ was located next to *TRPC4AP* and contained miR-499, whereas *MYH*_*M3383*_ was located next to sulfatase 2 gene (*SULF2*) and did not contain miR-499 in its intron. In tetrapods, however, *SULF2* is located in the same chromosome as *MYH14*/miR-499, but far from the locus. Based on the synteny, two putative *MYH14s*, one containing miR-499 and the other lacking it, were also found in green spotted puffer *Tetraodon nigroviridis* and tilapia *Oreochromis niloticus*. Interestingly, in Atlantic cod *Gadus morhua*, stickleback *Gasterosteus aculeatus*, platyfish *Xiphophorus maculatus*, and medaka, miR-499 was present within the expected syntenic region that contained *TRPC4AP*, *NDRG3*, *SULF2.* However, *MYH14* was absent in each case. Cod and stickleback retained a single *MYH14* paralog lacking miR-499 in the other syntenic region that contained *SULF2*. *SULF2* seems to be consistently located next to *MYH14* in most teleost fish species. Interestingly, the medaka genome was lacking *MYH14*. Although we screened the *MYH14* sequence from the Ensembl medaka genome and medaka EST data sets deposited to DDBJ/EMBL/GenBank using tBLASTn and the torafugu MYH14-1 (MYH_M5_) protein sequence as a query, no *MYH14* sequence was retrieved.

**Table 1:** Gene IDs of MYH14 and miR-499 used in this study

**Source**	**Gene**	**Database**	**ID**	**Position**	
Atlantic cod	*MYH14*	Ensembl	ENSGMOG00000012704	GeneScaffold92	21379-49726bp
Coelacanth	*MYH14*	Ensembl	ENSLACG00000018133	JH126566.1	2784820-286153bp
Green spotted puffer	*MYH14-1*	Ensembl	ENSTNIG00000007310	Chr11	10013139-10024708bp
Green spotted puffer	*MYH14-2*	Ensembl	ENSTNIG00000005523	Un_random	78966693-78979708bp
Stickleback	*MYH14*	Ensembl	ENSGACG00000000239	scaffold_114	38137-54868bp
Tilapia	*MYH14-1*	Ensembl	ENSONIG00000020306	GL831152.1	3238601-3253324bp
Tilapia	*MYH14-2*	Ensembl	ENSONIG00000014700	GL831175.1	1378557-1411608bp
Torafugu	*MYH14-1(MYHM5)*	Ensembl	ENSTRUG00000011087	scaffold_79	625079-637153bp
		DDBJ/EMBL/GenBank	AB235127		
Torafugu	*MYH14-2(MYHM3383)*	Ensembl	ENSTRUG00000002856	scaffold_398	58536-75364bp
		DDBJ/EMBL/GenBank	AB235128		
Zebrafish	*MYH14-1*	Ensembl	ENSDARG00000076075	Chr11	26116668-26147204bp
		DDBJ/EMBL/GenBank	JN216840		
Zebrafish	*MYH14-2*	Ensembl	ENSDARG00000035322	Chr23	18893433-18927796bp
Zebrafish	*MYH14-3*	Ensembl	ENSDARG00000094982	Chr23	19118523-19145602bp
Platyfish	*MYH14*	Ensembl	ENSXMAG00000003166	Scaffold JH556788.1	1,000,495-1,020,944
Spotted gar	*MYH14*	Pre ensembl	-	LG18	9021426-9045727bp
Chicken	*MYH14*	Ensembl	ENSGALG00000003157	Chr20	2584788-2613111bp
Human	*MYH14*	Ensembl	ENSG00000078814	Chr20	33563206-33590240bp
Atlantic cod	miR-499	Ensembl	ENSGMOG00000021764	GeneScaffold2467	713672-713752bp
Coelacanth	miR-499	Ensembl	ENSLACG00000021819	JH126566.1	2848699-2848788bp
Green spotted puffer	miR-499	Ensembl	ENSTNIG00000020076	Chr11	10019969-10020055bp
Medaka	miR-499	Ensembl	ENSORLG00000020982	Chr5	26842790-26842873bp
Stickleback	miR-499	Ensembl	ENSGACG00000021407	GroupXVII	10484744-10484827bp
Tilapia	miR-499	Ensembl	ENSONIG00000021569	GL831152.1	3243791-3243884bp
Torafugu	miR-499	Ensembl	ENSTRUG00000018774	Scaffold79	632933-633021bp
Zebrafish	miR-499-1	Ensembl	ENSDARG00000080181	Chr11	26138712-26138802bp
Zebrafish	miR-499-2	Ensembl	ENSDARG00000081473	Chr23	18902040-18902117bp
Zebrafish	miR-499-3	Ensembl	ENSDARG00000087228	Chr23	19122484-19122561bp
Platyfish	miR-499	Ensembl	ENSXMAG00000020681	Scaffold JH556712.1	243,217-243,307
Spotted gar	miR-499	Pre ensembl	-	LG18	9034506-9034572bp
Chicken	miR-499	Ensembl	ENSGALG00000021774	Chr20	2599334-2599424bp
Human	miR-499	Ensembl	ENSG00000207635	Chr20	33578179-33578300bp

### Phylogenetic analysis of MYH14 and miR-499

**Figure 2:**
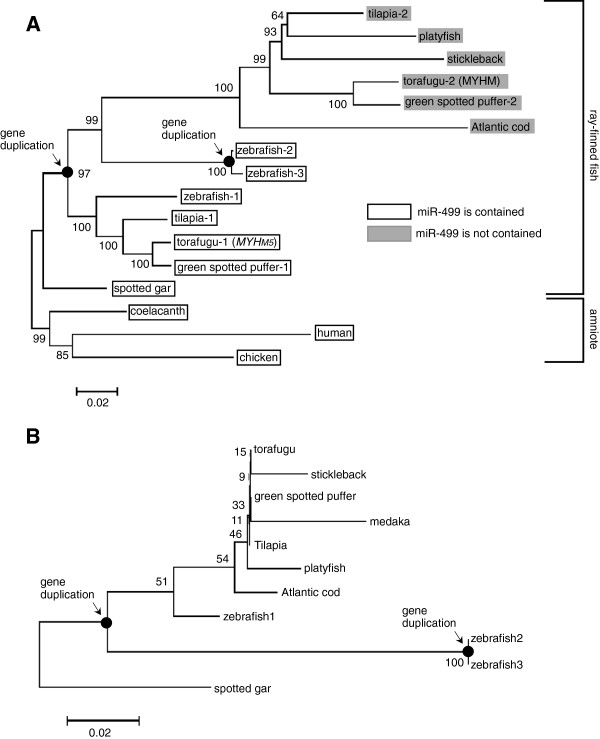
***MYH14 *and miR-499phylogenetic analysis.***MYH14***(A)** and miR-499 **(B)** neighbor-joining (NJ) trees. Bootstrap values from 1000 replicate analysis are given at the nodes as percentage values. Black circles indicate duplication of the *MYH14/*miR-499 locus.

Phylogenetic analyses based on the *MYH14* coding and miR-499 stem-loop sequences were performed to clarify the evolutionary history of the *MYH14*/miR-499 locus in teleost fish. Figure 2A and Additional file 1: Figure S1A show neighbor-joining (NJ) and maximum-likelihood (ML) trees of the *MYH14*s*.* Both trees show almost the same phylogenetic relationship, indicating the reliability of the phylogenetic relationships observed in this study. *MYH14* was monophyletic in the amniote lineage, including humans, chickens, and coelacanths, but was duplicated in the ray-finned fish lineage, except for the spotted gar (Figure 2A). Therefore, both *MYH14*s in teleost fish are paralogous genes that diverged at the base of neoteleostei lineage. *MYH14* paralogs were separated, except for zebrafish, according to the presence or absence of miR-499 in their introns. Note that accelerated evolution was clearly observed in *MYH14*s lacking miR-499 by their large genetic distance from *MYH14* possessing miR-499, suggesting a functional relationship between *MYH14* and miR-499.

The miR-499s phylogenetic relationships (Figure 2B and Additional file 1: Figure S1B) were consistent with those of the *MYH14*s. Although the bootstrap value in each node was quite low, three zebrafish miR-499 paralogs, miR-499-1, -2, and −3, were divided into two clades. Zebrafish miR-499-1 formed a single cluster with other teleost fish miR-499s.

The combined phylogenetic and synteny analyses suggest that the *MYH14*/miR-499 locus was duplicated early in teleost evolution and one of the duplicated miR-499 genes was lost in the common ancestor to cod and the Acanthopterygii, after the split from the zebrafish lineage. Additionally, *MYH14*s have seemingly been lost at independent points of teleost evolution.

### miR-499 expression in medaka

**Figure 3:**
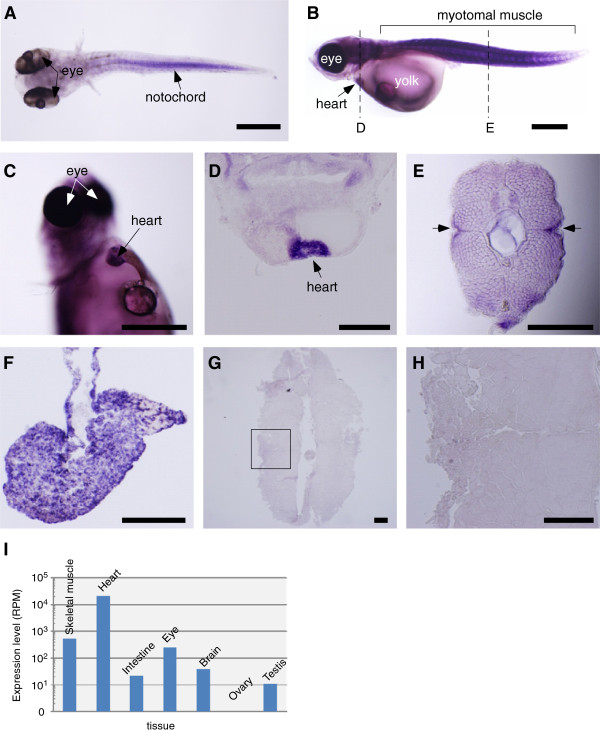
**miR-499 expression in medaka.** Whole mount of a medaka embryo at 5 days post fertilization (dpf) **(A)** and a hatching larva at 10 dpf **(B)**. miR-499 transcripts were detected in the notochord of the embryo and in cardiac and trunk skeletal muscles in the hatching larva. **C)** Ventral view of miR-499 expression in the heart of a 10-dpf larva. **D)** Transverse section of cardiac muscle at the position indicated in panel **B**. **E)** Transverse section from trunk skeletal muscle at the position indicated in panel **B**. Arrows indicate miR-499 expression in superficial slow muscle fibers. Transverse sections of adult cardiac **(F)** and trunk skeletal muscles **(G)**. **H)** Higher magnification of the square indicated in panel **G**. miR-499 was expressed in cardiac but not in trunk muscle at the adult stage. **I)** miR-499 expression confirmed by next-generation sequencing. Vertical axis indicates miR-499 read numbers in each tissue. Scale bars: **A**-**C**, 500 μm; **D**-**H**, 200 μm.

To find out whether miRNA-499 can be expressed despite lacking its host gene, its expression in medaka was examined by *in situ* hybridization and next-generation sequencing. We observed that medaka miR-499 was expressed at the embryonic stage in the notochord (Figure 3A), miR-499 expression in the notochord has not been previously reported in other animals. At the hatching stage, miR-499 was expressed in cardiac and trunk skeletal muscles (Figure 3B, C). The transverse sections of the medaka larva clearly showed miR-499 expression in the heart (Figure 3D) and the lateral surface of the myotomal muscle (Figure 3E) where slow muscle fibers are present. These expression patterns are consistent with those of their mammalian and zebrafish counterparts. To localize miR-499 transcripts in adult medaka, *in situ* hybridization was performed with transverse sections of trunk skeletal and cardiac muscles. Unlike the embryonic and larval stages, the adult medaka only exhibited strong miR-499 expression in the cardiac muscle (Figure 3F-H). This miR-499 expression pattern in the adult stage was also confirmed by next-generation sequencing (Figure 3I). Although miR-499 was detected in the adult medaka tissues examined, much higher miR-499 reads were obtained from the cardiac muscle (reads per million [RPM] = 20,624) when compared with skeletal muscle (544), eye (256), brain (40), intestine (22), testis (11), and ovary tissues (0) (Figure 3I).

### Sequence analysis of MYH14/miR-499 locus flanking regions

**Figure 4:**
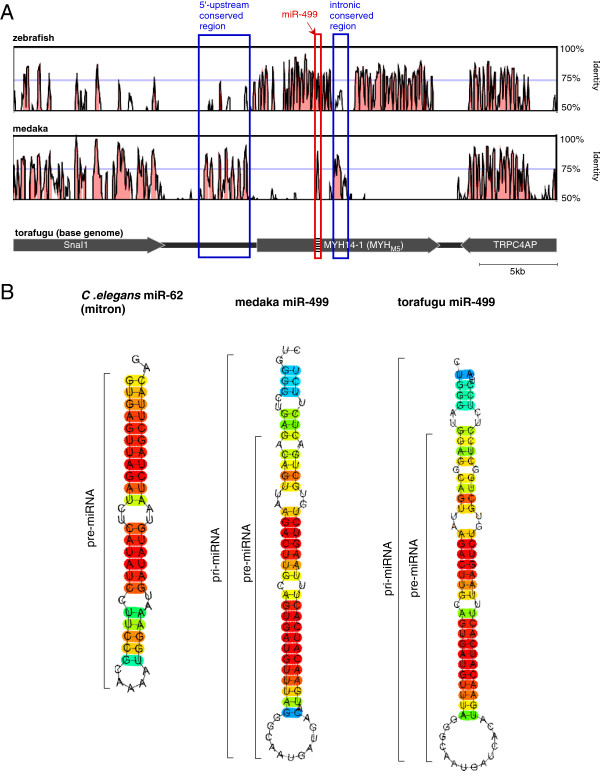
**Medaka miR-499 characteristics. (A)** Comparison of the flanking and related sequences of torafugu *MYH14-1* (*MYH*_*M5*_) with zebrafish *MYH14-1* and medaka miR-499. Highly conserved (>75%) regions between the two sequences are indicated by red-shaded peaks. Several highly conserved regions were identified at the *MYH14*/miR-499 5′-flanking and intron, as shown in blue boxes. **(B)** Putative secondary structures of mirtron (*Caenorhabditis elegans* miR-62) and miR-499.

Intronic miRNAs can be independently transcribed from their host gene by using their own promoter positioned immediately upstream of miRNAs [19]. For medaka, miR-499 is transcribed lacking its host gene *MYH14*, which suggests the presence of its own promoter for transcription. Figure 4A shows comparisons of torafugu *MYH14-1* (*MYH*_*M5*_) flanking regions with corresponding regions in zebrafish *MYH14-1* and medaka miR-499. In the case of medaka, *MYH14* was completely absent, with the exception of miR-499 (Figure 4A and Additional file 2: Figure S2) and an intron immediately downstream of miR-499 (intronic conserved region in Figure 4A, Additional file 3: Figure S3). Interestingly, the torafugu and zebrafish *MYH14s* 5′-flanking sequences showed clear similarity with those of medaka miR-499 (5′-upstream conserved regions in Figure 4A, Additional file 4: Figure S4). Although the conservation in the zebrafish *MYH14-1* 5′-flanking region was not so obvious, it still contained several highly conserved regions (Additional file 4: Figure S4).

### Secondary structure of the miR-499 stem-loop sequence

Intronic miRNA is transcribed as pre-mRNA from a part of an intron in the host gene [20]. miRNA endowed by an intron folds to form a local double-stranded stem-loop structure called the primary miRNA (pri-miRNA). In animals, RNase III drosha crops pri-miRNA at the stem-loop during splicing and produces a precursor miRNA (pre-miRNA), which is then processed by dicer to form mature miRNA. From these canonical intronic miRNAs, a new type of intronic miRNA called mirtron has been discovered. Mirtrons are embedded in short introns, and their biogenesis does not require drosha cropping. The pre-miRNA of mirtron is produced directly by splicing [21-23]. Figure 4B shows miR-499 predicted stem-loop structures from medaka, torafugu, and the representative mirtron, miR-62, from *Caenorhabditis elegans*. miR-499s have longer stem-loop regions than those of mirtrons and are processed by drosha to produce pre-miRNAs. The torafugu *MYH14* intron containing miR-499 is 247 bp in length (see Additional file 2: Figure S2), which is long enough to produce canonical miRNA hairpins to be cut by drosha. These results combined suggest that miR-499 is not a mirtron but a canonical intronic miRNA. However, experimental proof is required to confirm whether miR-499 requires drosha processing.

## Discussion

**Figure 5:**
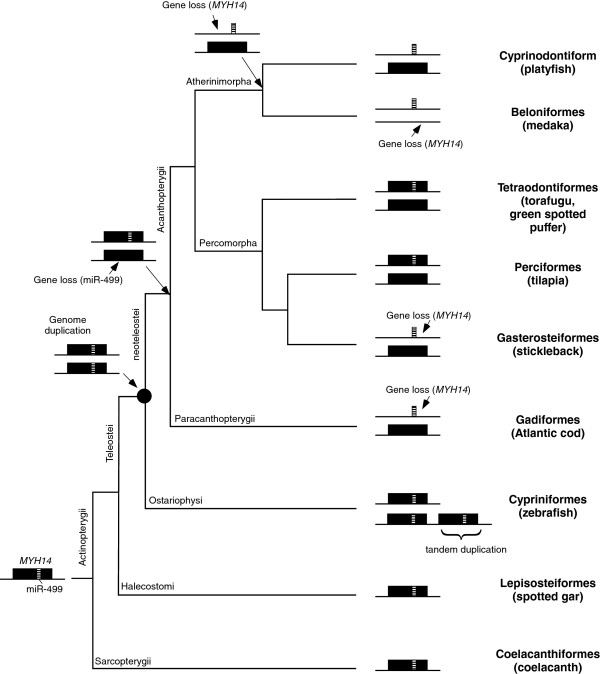
**Putative evolutionary history of *MYH14 *and miR-499 in the fish lineage.** The common ancestor of amniotes and fish had a single miR-499 containing *MYH14*. Neoteleostei-specific whole genome duplication formed two sets of *MYH14*/miR-499 pairs. In the zebrafish lineage, additional tandem duplication resulted in three *MYH14*/miR-499 pairs. In torafugu, green spotted puffer, and tilapia, redundancy in miR-499 caused the deletion of one of the two miR-499 paralogs. In the stickleback and Atlantic cod lineage, an additional gene loss occurred in one of the two *MYH14* paralogs and loss of the remaining *MYH14* gene resulted in its complete elimination from the medaka genome.

Figure 5 shows the putative evolutionary history of the *MYH14*/miR-499 locus in teleost fish. It has been proven that after two rounds of whole genome duplication (WGD) in a common ancestor of vertebrates, a third WGD occurred in the fish lineage [24-28]. This fish-specific WGD occurred at the base of the Teleostei lineage, after diverging from ancient fish groups such as Polypteriformes, Acipenseriformes, and Lepisosteidae [29]. Our phylogenetic analysis clearly shows duplication of the *MYH14*/miR-499 locus after the divergence of spotted gar, indicating that the teleostei-specific WGD provided present-day *MYH14*/miR-499 paralogs in teleost fish. *TRPC4AP* and *SULF2* genes located next to *MYH14*, were also duplicated in the fish-specific WGD. However, information on Osteoglossomorpha, Elopomorpha, Clupeomorpha, and Protacanthopterygii, which are important fish groups comprised of neoteleostei, was not reviewed in this study. Therefore, further analysis is required to fully reveal *MYH14*/miR-499 evolution in fish.

The existence of multiple *MYH14* and miR-499 genes in various teleost fish suggests their expressional and functional versatilities. Torafugu *MYH14-1* (*MYH*_*M5*_) expression was observed in both slow and cardiac muscles in the developmental and adult stages, whereas *MYH14-2* (*MYH*_*M3383*_) expression was restricted to adult slow muscle [13,14]. Zebrafish *MYH14-1* was expressed in both slow and cardiac muscles in the early developmental stages and in slow and intermediate muscles in the adult stage [15]. Furthermore, our present study demonstrates that medaka miR-499 expression differed from the above-mentioned *MYH14*expression patterns (see Figure 3). It would be interesting to determine whether such differences in *MYH14* and miR-499 are related to physiological and ecological variations among teleost fish species. Fish are the most diverse vertebrate group consisting of over 22,000 species. In response to the wide range of environmental and physiological conditions they encounter, the characteristics of fish musculature, including muscle fiber-type composition, are also highly diverse. Medaka makes a particularly interesting subject because of the complete elimination of *MYH14* from its genome. Although muscle fiber-type composition has not been well characterized in medaka, Ono et al. [30] reported an *MYH* gene specifically expressed in slow muscle fibers at the horizontal myoseptum. Such *MYH* expression has never been reported in other teleost fish species. In contrast, medaka fast muscle exhibits high plasticity to adapt to temperature fluctuations by changing *MYH* expression [18,31]. Further comparative analyses of *MYH14* and miR-499 may shed light on the mechanisms involved in the formation of species-specific musculature evolution.

The loss of the intronic miRNA in the ancestor of cod and the Acanthopterygii might be explained by functional redundancy. The loss of intronic miRNA from the host gene is possible if mutations are introduced into an intron without any effect on the function and expression of the host gene. Stickleback, medaka, and Atlantic cod display the opposite pattern with the intronic miRNA lacking its host gene. Intronic miRNAs are transcribed with their host genes, and thus, coordinated expression between an intronic miRNA and its host gene is frequently observed [32]. In the present study, however, medaka miR-499 was actually expressed in various tissues despite the absence of *MYH14* (see Figure 3). How does intronic miRNA remain after the loss of its host gene? We speculate that miR-499 is a canonical intronic miRNA produced by drosha cropping (see Figure 4B). Recent studies have revealed that splicing and pre-miRNA cropping by drosha are independent processes, indicating that splicing is not essential for intronic miRNA production [33]. In other words, severe mutations of the host gene may not affect the production of intronic miRNAs in the presence of the host gene transcriptional system. Interestingly, sequence comparison analysis showed highly conserved 5′-flanking regions between torafugu *MYH*_*M5*_ and medaka miR-499 (see Figure 5A). The spatio-temporal expression of the major skeletal *MYHs* in teleost fish is regulated by small regions scattered throughout the 5′-flanking sequence [18,30,34,35]. Recently, Yeung et al. [36] reported promoter activity in a 6.2-kb upstream sequence of mouse *MYH14* that mimics endogenous *MYH14* and miR-499 expression. Therefore, these conserved regions in the 5′-flanking sequence may act as a promoter for the spatio-temporal expression of *MYH14*, and the regulatory sequences are conserved in medaka miR-499 despite the loss of the *MYH14* gene. We could also speculate that miR-499 has its own promoter as do some intronic miRNAs. In fact, Matthew et al. [37] reported uncoupled *MYH14* and miRNA-499 expression in mice, suggesting the independent transcriptional regulation of miR-499 from *MYH14*. Isik et al. [38] found a conserved region immediately upstream of some intronic miRNAs in *C. elegans* and demonstrated in promoter activity the conserved region. An intronic sequence immediately downstream of miR-499 is conserved among zebrafish, torafugu, and medaka, as shown in Figure 4A, which could be the miR-499 promoter. These findings can potentially explain why miR-499 has remained despite the loss of *MYH14* in some teleost fish genomes. To our knowledge, this is the first report that describes the conversion of intronic into non-intronic miRNA during evolution. Comparative analysis of transcriptional regulation between intronic and intergenic miR-499s will provide new insights into miRNA evolution.

## Methods

### Fish

All procedures in this study were performed according to the Animal Experimental Guidelines for The University of Tokyo. Live adult medaka specimens (average body weight of 0.78 g) were reared in local tap water with a circulating system at 28.5°C under a 14:10-h light–dark photoperiod, at a fish rearing facility in the Department of Aquatic Bioscience, The University of Tokyo. Tissue for RNA extraction was dissected after instant euthanasia by decapitation and stored in RNAlater (Invitrogen, San Diego, CA, USA). Embryos were obtained by natural spawning and raised at 28.5°C. The developmental stage was determined by the number of days post fertilization.

### Construction of a physical map around MYH14 and miR-499

The Ensembl genome browser (http://www.ensembl.org/index.html) was used to determine the syntenic organization in the region surrounding *MYH14* and/or miR-499 in vertebrates. The database versions used were as follows: human (GRCh37), chicken (Galgal4), coelacanth *L. chalumnae* (LatCha1), zebrafish *D. rerio* (Zv9), torafugu *T. rubripes* (FUGU4), green spotted puffer *T. nigroviridis* (TETRAODON8), tilapia *O. niloticus* (Orenil1.0), Atlantic cod *G. morhua* (gadMor1), stickleback *G. aculeatus* (BROADS1), platyfish *X. maculatus* (Xipmac4.4.2), and medaka *O. latipes* (MEDAKA1). The pre Ensembl browser (http://pre.ensembl.org/index.html) was used for analysis of spotted gar *L. oculatus* (LepOcu1).

### Bioinformatics analysis

The *MYH14* and miR-499 sequence data were retrieved from the available genome databases mentioned above (Table 1). NJ and ML trees were constructed on the basis of the *MYH14* coding and miR-499 stem-loop sequences using MEGA5 [39] with 1000 bootstrap replications. The Nei and Gojyobori method [40] (Jukes-Cantor) was employed to consider synonymous and non-synonymous substitutions for the *MYH14* NJ tree. The Tajima-Nei model [41] was employed for the miR-499 NJ tree, whereas the Tamura-Nei model [42] was used for the *MYH14* and miR-499 ML trees. The torafugu *MYH14-1* (*MYH*_*M5*_), zebrafish *MYH14-1* 5′- and 3′-flanking sequences, and the medaka miR-499 stem-loop sequences, which contain *Snai1* and *TRPC4AP* genes, were retrieved from the Ensembl genome browser. The homology search on the flanking sequences was carried out using the mVISTA alignment program through the vista server (http://genome.lbl.gov/vista/index.shtml). Putative secondary structures of the miR-499 from medaka and torafugu stem-loop sequences and that of the *C. elegans* mirtron miR-62 (miRBase accession number: MI0000033) were predicted using the RNA fold program CentroidFold (http://www.ncrna.org/centroidfold).

### Small RNA library construction and sequencing

Total RNA was extracted from the muscle, intestine, eye, brain, heart, ovary, and testis of adult medaka using a mirVana™ miRNA Isolation Kit (Applied Biosystems, Foster City, CA, USA). Small RNAs (less than 40 nucleotides in size) were purified from total RNA using a flashPAGE™ Fractionator (Applied Biosystems), and the small RNA libraries were constructed according to the manufacturer’s instructions. Library sequencing was performed with SOLiD™ next-generation sequencer (Applied Biosystems). After elimination of low-quality reads using perl scripts of our own design, 102, 602, 452 reads of 35 nucleotides were obtained. The 18–25 nucleotide reads were subjected to a Blast search against known mature miRNA sequences deposited in miRBase 18.0 (http://www.mirbase.org/). The sequences with their seed regions (2–8 nucleotides from the 5′-end) showing 100% identity to those of known mature miR-499 sequences were annotated as medaka miR-499. Gene expression was represented as reads per million (RPM), which corresponds to (total reads of a given gene/total reads in the tissue) × 10^6^. Sequence data sets used in this study were deposited at the DDBJ Sequence Read Archive under the accession number DRA001039 and DRA001040.

### In situ hybridization

We used a digoxigenin (DIG)-labeled MiRCURY detection probe (Exiqon, Copenhagen, Denmark), an LNA-modified oligo DNA probe containing the miR-499 mature sequence (5′-AAACATCACTGCAAGTCTTAA-3′), to detect miR-499 transcripts. *In situ* hybridizations were performed according to Kloosterman et al. [43]. The adult, embryo, and larval medaka trunk skeletal and cardiac muscles were fixed in 4% PFA at 4°C overnight. Transverse sections of the tissues were cut at 16-μm thickness. All hybridizations were performed at 66°C, which was 20°C below the predicted melting temperature (Tm) of the LNA probe. Alkaline phosphatase-conjugated anti-DIG antibody (Roche Diagnostics, Penzberg, Germany) and nitroblue tetrazolium chloride/5-bromo-4-chloro-3-indolyl phosphate were used for signal detection with an MVX10 stereomicroscope (Olympus, Tokyo, Japan).

## Competing interests

The authors have no financial or other competing interests to declare.

## Authors’ contributions

B.S.S. and K.S. were involved in the conception and design, and data acquisition and interpretation. W.C. carried out next-generation sequencing data retrieval and analysis, and A.M. assisted in fish breeding and data analysis. A.S. and S.W. participated in research design, coordination, and helped to draft the manuscript. All authors have read and approved the final manuscript.

## Supplementary Material

Additional file 1: Figure S1 *MYH14* and mIR-499 phylogenetic analysis. *MYH14* (A) and miR-499 (B) maximum-likelihood (ML) trees. Bootstrap values from 1000 replicates analysis are given at the nodes as percentage values. Click here for file

Additional file 2: Figure S2 Sequence comparison of the intron containing miR-499 among torafugu, zebrafish, and medaka. Shaded sequences are highly conserved regions among the three fish species. Mature miR-499 sequences are boxed. Bold letters indicate 5′ and 3′ intron splice sites. Numbers on the right indicate the positions of the *MYH14* (torafugu and zebrafish) start codon and mature miR-499 (medaka) 5′-end. Nucleotide sequences were aligned by CLUSTALW. Click here for file

Additional file 3: Figure S3 Intronic conserved regions in *MYH14* among torafugu, zebrafish, and medaka. The red box shows highly conserved regions among the three fish species. Bold letters indicate 5′ and 3′ splice intron sites. Numbers on the right indicate the positions of the *MYH14* (torafugu and zebrafish) start codon and mature miR-499 (medaka) 5′-end. Nucleotide sequences were aligned by CLUSTALW. Click here for file

Additional file 4: Figure S4 5′-flanking conserved regions in *MYH14* among torafugu, zebrafish, and medaka. The red and gray boxes show highly conserved regions between torafugu and medaka, and among the three fish species, respectively. Bold letters indicate 5′ and 3′ splice intron sites. Numbers on the right indicate the positions of the *MYH14* (torafugu and zebrafish) start codon and mature miR-499 (medaka) 5′-end. Nucleotide sequences were aligned by CLUSTALW. Click here for file
