# Supplementary figures and images for: Evolution of the myosin heavy chain gene MYH14 and its intronic microRNA miR-499: muscle-specific miR-499 expression persists in the absence of the ancestral host gene
Source: BMC Evol Biol. 2013 Jul 6;13:142. doi: 10.1186/1471-2148-13-142 (PMC3716903; doi:10.1186/1471-2148-13-142)

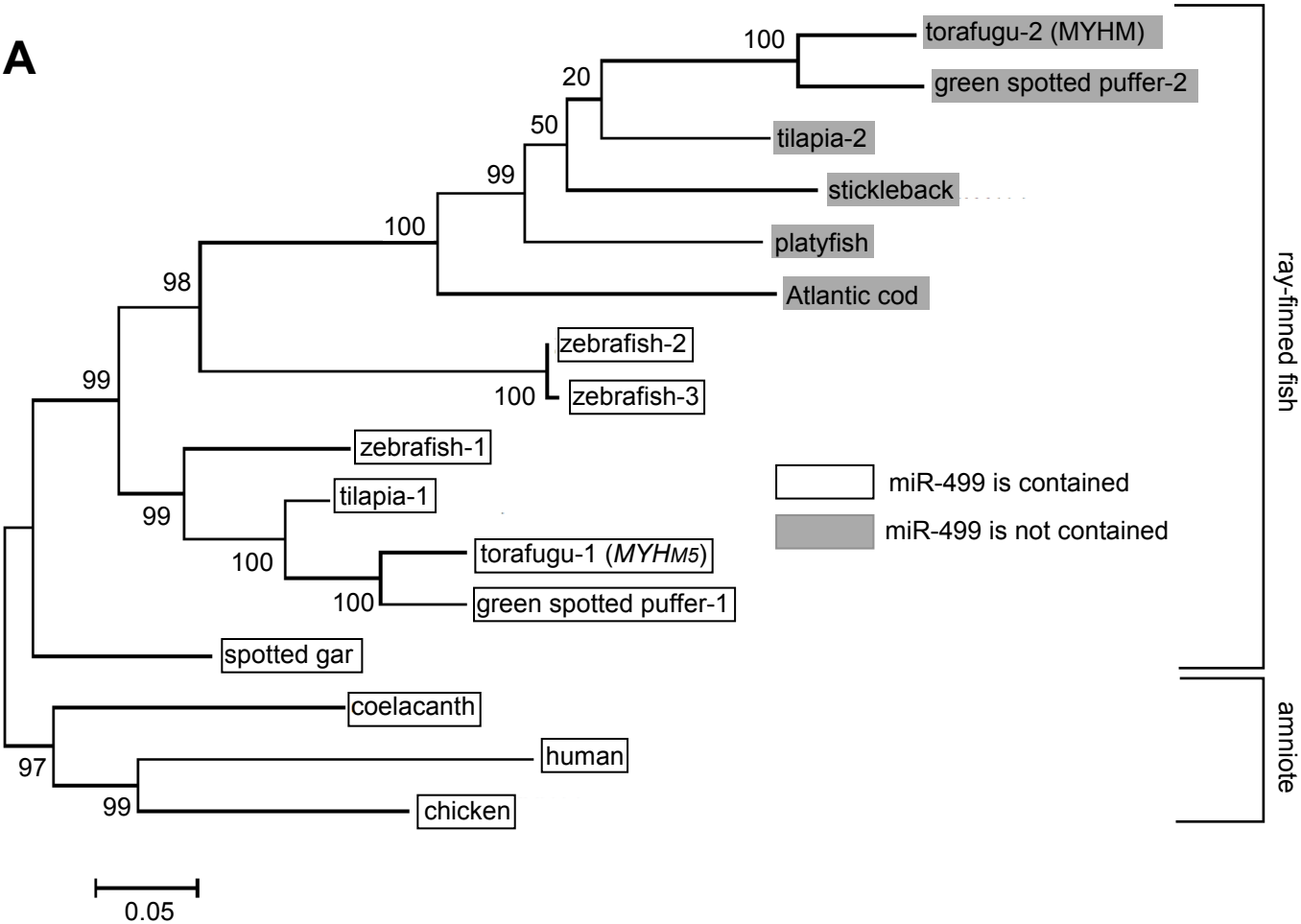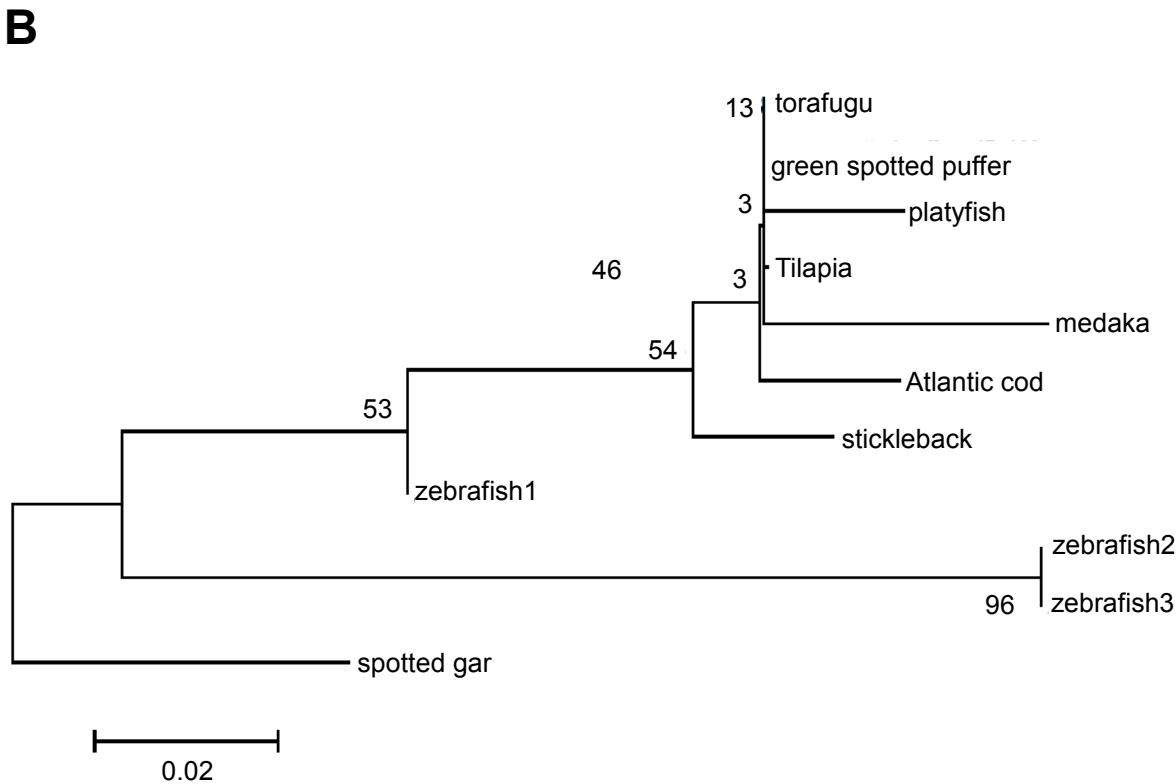

Supplement: Additional file 1: Figure S1 — MYH14 and mIR-499 phylogenetic analysis. MYH14 (A) and miR-499 (B) maximum-likelihood (ML) trees. Bootstrap values from 1000 replicates analysis are given at the nodes as percentage values. [file 1471-2148-13-142-S1.pdf]
