# Supplementary material for: Evolution of the myosin heavy chain gene MYH14 and its intronic microRNA miR-499: muscle-specific miR-499 expression persists in the absence of the ancestral host gene
Source: BMC Evol Biol. 2013 Jul 6;13:142. doi: 10.1186/1471-2148-13-142 (PMC3716903; doi:10.1186/1471-2148-13-142)
Supplement: Additional file 2: Figure S2 — Sequence comparison of the intron containing miR-499 among torafugu, zebrafish, and medaka. Shaded sequences are highly conserved regions among the three fish species. Mature miR-499 sequences are boxed. Bold letters indicate 5′ and 3′ intron splice sites. Numbers on the right indicate the positions of the MYH14 (torafugu and zebrafish) start codon and mature miR-499 (medaka) 5′-end. Nucleotide sequences were aligned by CLUSTALW. [file 1471-2148-13-142-S2.pdf]

(Sharmin et al., Supplementary Figure 2)

```
  torafugu GTGAGAGGGAGGTCGGAACGGGGGTCGAATGTTGACGCGTGCTGTCCGAAGTTCTGAGT----------------------------GGGAAAA------ 4130
zebrafish GTGG------GACTTTAACCAGACTTCACTACTGT---TTTCAGTAGATTATTGATATTAAAAAAAACTATTATCTGGGGTCGTTAAAAGAAAATGAAAA 8393
    medaka GTAAAGGTCATTTAGGTACCGGACCTGAATGTGGTCGCTTTCTGACCATCAGTCAGCGCACAGAGAGCCGTCA--------------GAGGACA------ -16

                                         mature miR-499
  torafugu ---------GCTGGGATGGAGGCAGTTAAGACTTGCAGTGATGTTTAGGGCAATGATCACATGAACATCACTTTAAGTCTGTGCTGGCTCCTCT------ 4215
zebrafish CTGTCCAATACTGAGAGGGAGGCAGTTAAGACTTGCAGTGATGTTTAGAGAAATG-TCACATGAACATCACTTTAAGTCTGTGCTGGCTCCTGTTCTGAG 8492
    medaka ---------GCTGGGGCTGAGACAGTTAAGACTTGCAGTGATGTTTAGGGCAATGATGACATGAACATCACTTTAAGTCTGTGCTGACTCTTCT------ 69

  torafugu -----------CCTCATCACGCCAAGG--------------------------------------ACGTC-ACCTG------------------------ 4241
zebrafish TCTACAAAGTGCTTCAACAACTCAAGGTAGGTCTAGAAATAACTGACAAAGTTAAAAAGTTAGACATTTTTACATGTTATGATCAATAGCATTTGGTTTG 8592
    medaka -----------CTTTTTCACGACGGGA--------------------------------------TTTTCTGCATG------------------------ 96

  torafugu --------TGAGGAGATCCAAATCCACC---------AGCAGCTTTATTTCTGTGCTGGGTGACTGATTGCTTC-TCTGACGCTGCAG 4311
zebrafish TTCAGTTTTAAAAAGATAAGAACTGATCTACAACAGTAATAGTTTTTGGTCTTGATTGTGTGCTTGATTGCTTCTCCTCCAACTATAG 8680
    medaka --------CGAGAGAGCCATGGCCGAGC---------AGAGGGTTTCT-----------GTGGGCATTTACCTC-CATAAATAAAATG 154
```
