# Supplementary material for: Evolution of the myosin heavy chain gene MYH14 and its intronic microRNA miR-499: muscle-specific miR-499 expression persists in the absence of the ancestral host gene
Source: BMC Evol Biol. 2013 Jul 6;13:142. doi: 10.1186/1471-2148-13-142 (PMC3716903; doi:10.1186/1471-2148-13-142)
Supplement: Additional file 3: Figure S3 — Intronic conserved regions in MYH14 among torafugu, zebrafish, and medaka. The red box shows highly conserved regions among the three fish species. Bold letters indicate 5′ and 3′ splice intron sites. Numbers on the right indicate the positions of the MYH14 (torafugu and zebrafish) start codon and mature miR-499 (medaka) 5′-end. Nucleotide sequences were aligned by CLUSTALW. [file 1471-2148-13-142-S3.zip › 1565208304857766_add3/1565208304857766_add3a.pdf]

```
 torafugu GTAAAAAT--------------AGGTCAAG---------------------------------------------------------------------------------- 5035
zebrafish GTATGAGCTAAAGATGTTTTAAGATTAAATCACAGCAGCCCCCATATATTTAGACATTCACTGCACACTTGAAAAAGAATGAAAGTCATTACATAAGATTGCAT 11255
   medaka GTATGAAC--------------AAAGTAAA---------------------------------------------------------------------------------- 476

 torafugu -------------------------------------------------------------------------------------------------------GTGGC 5040
zebrafish TTCAAACCAGCCTCCGTTCATTTTACAGAAAGCTATATTTTAAGTATGCTTGAAAACTAAACTAATCTATAGATTTTTTTTGCTCACATTGCTAGTTTTGTGGT 11360
   medaka -------------------------------------------------------------------------------------------------------GAGGA 715

 torafugu -----TTTAATCTTTGTCCAGAAATTCCTTT-----------------------------------------------------------------------TTTAAAAG 5074
zebrafish GAACAACTCATCTGTGTGAATAGTTTCCTCTTGTAATCTTTAATTAGAATCTGAAATCTTTAATTGAAACACTTCCTGTTTGTTTAAATTCTATCCATTTGAGGT 11465
   medaka AGACGTTTCCTCCTTTTGCTG-------TTT-----------------------------------------------------------------------TTTGTGGT 747

 torafugu CCTA------------------------------AATGAATAAAGGAAGTATTTCTAGATGCGGG---------------------AAAGCCTTT----------- 5118
zebrafish ATTGTCAACTGTCAGTTTTGACAGTGGTACGACAACAAACAAAAATGGTGAG--TAGGTGTTTGTTAGGTAGTAATAATTCTCCTAAAACCTTTTCCCAATTTTT 11568
   medaka GTTG------------------------------AAAGAGTGGAAAATGTGGGGTTGGGTGCAGG---------------------AAAGTCCTT----------- 791

 torafugu ---GAACATAACCCCGCGCTGGAAGGTGAAAACACGAGTTCTTAAAAAAAAAAAAAAAGCCTCCCCCGAAG------------------------------------ 5185
zebrafish CTGAATGAACTGCCTGCTTTACTAGATACAGTC---AGCTTGCAGTAGAAAAAAACAAGCCACCCCACAGATTTCTCATTTAATATTCCATTTCCTTAACTGCAT 11670
   medaka ----------------------------------GAGCTCTC----------AGAAGGTCCTCCTGCAC------------------------------------ 816

 torafugu --------------------------------------------CAGTTGTTTTTCTGAACAGGTTC------------------------------------ 5208
zebrafish CACAATACAAAAAAAGATTGCAACTTCTGGTTCATGGAGATTTTAACAGTAATATGTCTTTTTAGGTTTTCCAATTGTATGTTAAAAATAAATATGGATTACAAA 11775
   medaka --------------------------------------------CAGTTGCTTCTCCCAACAGGTTT------------------------------------ 839

 torafugu -------------------------------------------------------------------------------------------------------- 5208
zebrafish AGACTAAAACAAAAACACAACAAAAAGATTTAAGATTTTTTTGGAATATAGAATATAAAGATTACATATTCGGCTTTAGGTGGCAGCCGCAAGCTAAATTTCAC 11880
   medaka -------------------------------------------------------------------------------------------------------- 839

 torafugu ---------------------AATAGCAACACGATCA-------------------------------------GCTGCGAGTTTTTCAGCGTG--------- 5244
zebrafish AGGCTAACATGATAATTTTTGAATAGCACTGCTCACACAGAAGTCTGTTTCACACTGGGCAATTTCTGTTCCCATCGCTGTCAATTTTTGTGACTGACCTAAACC 11985
   medaka ---------------------AAT---CCCACGCTCA-------------------------------------GC------CTTCCGGGCCTC--------- 866

 torafugu -------------------------------------------------------------------------------------------------------- 5244
zebrafish CAATGGTAAACCCATGAGTGAAATATTTCATCCGGTGTGGGTTCCTTCAGAAATGGCTTAATTTTGCTCACAAAAATGTGCCACTTTATCCTACTAACACCTTGT 12090
   medaka -------------------------------------------------------------------------------------------------------- 866

 torafugu ----------------------------------------------------------------------------------------------------TGATAC 5250
zebrafish GTCCAATCCTTTTATTCTGAGAACGCGCTTCCAAAACTCTTTGATTTCCATATTAGCCTGAAATAATGCAGTCCCACACAAATATTCACAAACTACATTGATAT 12195
   medaka ----------------------------------------------------------------------------------------------------TGATAC 872

 torafugu ACTATACCTTAGT----------------------------------GTTCGCTCCCTCGCTCCAGTATGTGCGGCATGCAAGGGCAGTGTTTTCGACGTGTGTG 5321
zebrafish CCAACAGGTTTGCTCCTAATAAAGTTAGCAACAATCTGTTGTCACATGATAGCTCTCAATCCTCAACATGAGAAGTATTTTGGAGTCATGTGGATGGAGTGTG 12300
   medaka ACTATAGCTTTGC----------------------------------ATTTACTGCCCTACCGGAACACGAGTGGCATGCAAGGGTAATGTTATCGGCGTGTGTG 943

 torafugu AGGCGTGTG--------CTTGCCCTTCTATGGTCACCAGGCATGTGTGTGGCCTCAACTCTTAATAAGAGCTCCACTCAACCTTCACACACGCACCACTCCTACA 5418
zebrafish AGGCGTGTGTCTTCAGACATGCATTTGT-------CCATGTGTATGTGTGGCCGAGACCCTTCAT--GCGCTCCGACACTTTCTTTCAAACACTC---------CA 12388
   medaka AGGCGTGTG--------CTTGCCCTTGTTTGGTTAGCGGGCATGTGTGTGGCC-CTGCCGTTAATAAGGCCTCAGTTTCTGCCGTGCACACATATCCCATTCGCA 1039

 torafugu GGTCTCTGGCCATTCTCGCCATTCACACTCTCTAAATTAAACAATGCCGATGGCGTCCTGCGGAGCCAGGTGGCGACATGTGTCAAGCAGCAGCTGC----CTCG 5519
zebrafish TGTCTATATCTATGATCTCCACCCTTTCCTTTTA--TTAGACAATGCAGGCGGTGTCCT-TAAAGCCCAGGGACCCTGTGGGTCACAAGGCAGCTGCTCACTCCA 12490
   medaka GGTCTCTGGCCATTCTCTCTCTACGCTCTTTCTAATTTCAACAATGCAGACGGTGTCCT-CAGAGCCAGGTGGCGACATGTGTCAAGTGGCAGCTG-----CTCA 1138

 torafugu GCCCTCCCTAAAAATACAACCCTCCCCCCCACTGC-----CCGATGGCCTCTGGCCCCTTTTGTCCATGGCGCCTGCACTTCTCAGTGAGTGCATTGATGGGATA 5619
zebrafish CTCCTCACTAAAAATACAGGCCAGCACCCTTCTCAAA---------------GGACAGTTTTG-CCAAGGCGCCTGAGCCTCTAAAGGACCCCATTGAAAGCATA 12579
   medaka GCCCACCCTAAAAATACAACTTCCCCCCCCTCCCTGGACACTGATGGCCCCTGGCCTCTTTTGTCCGCGGCGCCTATACTTCTAGGCAAATGCATTGATGGGATG 1243

 torafugu TGTGGCGGGTGACAGAGGATTTACGTGGGACGCCGGACAGAGGGCTTTGGTTTCTAAAT-AAACA-CAGACGC-TCACC-TTTGAGAGTCATGCAACACACAGCC 5720
zebrafish TCTGCAGACTGGCAAAGGATTAAAGCCTTTGGTCCAACAGAAGGCCTCGGTTTCTAAAATAAATATTAGGCACTTCACCTTTTGGGTGGCCTGGGGCACAGATGC 12684
   medaka CGTGGCGGGTGACAGAGGATTAAAGCCAGTGGGCTCAAAGGGGGCTTCAGTTCACAAATAAAACA-AAAATGC-TCACCTTTTTAGGGGCACCTGGTGTGGACTG 1346

 torafugu ATGTCAAAAATGTAATCAGCCCCGCCACCCACCTCCCCCGACCCATGCAGCCCGCCTCCCTATCTTGTGTGGACACTTACGCTCAGGCTTTTAACATTGACGGGAA 5825
zebrafish ATGGCAGCTATTTGATCAGAC------------------------------CGAATTCTCTGCGTCAT-------------------CTGCTAACATGTAACCGCA 12741
   medaka AAG-AAAAAATGTGATGAG---------------GCCCCAACACA-GAGGCGGGCTCTC----------------------------CTTT-------------AA 1394

 torafugu GTCCTTGACCAGCGTGCTC-TGGGTCCAAATTTAGGCCCACGAGACACCATTCTC-CAGAGACCAAATCAGCC-TGGTCACTCTGCTCTTTCCCAGCCGTGAAAC 5927
zebrafish GTACTGTGACAGCAAGTGCATG-----------ACATCTGAAAGTCACC-------CCAGTAATAGACTAACTAAAATGAACCTCGTTTCAAGCATTCATGGA-- 12826
   medaka GTCCTTGACCATACTGTCCAAGGGTCCCTATTTACATCTGTGAGACCCCGTCCTCGCTGGGACTAAACCAACC-TGGCAAGCCTGTGTGGCTGCAGCCATGAAAC 1498

 torafugu TTCAAAAGCAGCAGCAGCAGGTTTA-------TGAT-------------TTCATGT-----------TGGAGGGGAT--GGTTGCA-----------GGATTA 5986
zebrafish --CATAAG-----CCAGTGGGTCTTATGTGGGTGATAGTTTTCCCATGGTCCGTGTTATTATTGATAATCAAAGCTATTCAATTACAAATTCACAAACCAAAGTG 12924
   medaka ATCAAA---AGCAGCGTTTGGTGTT-------TGAT-------------TTCATGTGATTAAATATAATAGGACTGGT--AGCTGCA-----------GAAGAA 1566

 torafugu GTGCACCAACACATTAAGCACCAAGGACAAGTAACTGCA-------------------TAAATAAATAGA----------TGGGGAAC-TCCCAC---------- 6051
zebrafish ATGCTAAAATAAAACAAAACATAAGTTTAGGTTGAAGTACAAAACCTGAAATACATTTCTAATTAATAGAATAATATTATTATAAAAAAATCCATTAATTTACAG 13029
   medaka GTG---CCAAAAATTAAGCAGCAAGGAAAAG----------------------------TCAATAAATAAA----------CGGGGAAAGTCCCAC---------- 1621

 torafugu ------------------------ATAAATCTTCCTGACCTTCTTCC---------CTTTACTTGATCTGAACTTCATTTCGCCTGTTTCAAAGACACCCTGACC 6123
zebrafish TTCATGTTATCTGTGATGCATGAGTTTTACATTTATG-TATGCTTTCAAA--------TCGCATTATATGACCTTCATCTCTGC---TCTGATAACTTTCTATAA 13122
   medaka ------------------------TTTCACCTCCATGACCCACTTTCAGTGAAGGTCCTGTCCTCATCGGAGAGCCGGTACAGCAAAAGTGGAAACG-------C 1695

 torafugu TTGTCGTCA---TTCCCGTTCTTTCATGTTGTAT--------------------------------------GTTGCTACACACGTGCTACA------------ 6174
zebrafish CTTTCTATAATGTTTTTAGGCTTAACCTCTGTTTTAATTAGATATTTTGAGTATTTTAAGTTTAAATTGTAAATTAAGACACAAAAACCAGATAAAGCCAGTATG 13227
   medaka TTGTTTTTACTTTTATCGAGGTTTCCTTCTGCCT--------------------------------------GCTAAT-CATAAGCAGGAAA------------ 1748

 torafugu --------------------------CTTTATACATAATTATCTGC----TTTATTATATTTTGATTACATAAAAAAGTGCTCTGAG----------------- 6231
zebrafish TTTCAGTCATTTTAAAATGTTTTTTCCTTCATATATAATTGTTTATAC--AATATGTTGTTTCAGACTACTAAAAACTGTCACTGAAAGTCGTTTTGTTTAGCTG 13330
   medaka --------------------------TTGTATCCATTTTTCTCAACAGAAATTATTCTTTT-------------------CCTTTAG----------------- 1790

 torafugu -------------------------------------------------------------------------------------------------------- 6231
zebrafish TAGTGTTTTCAACATTAGAATGTTAAAGTCTAATGTAATGCTAAAATATTATTGTCAGAGGTTCACATTATTTTTATAGTTTAAATATGAATGAATGATTCTAAA 13435
   medaka -------------------------------------------------------------------------------------------------------- 1790

 torafugu --------------------------------------------------------------------------------AGCGGCCTCT-------------ATCAAC 6247
zebrafish GATGTCACTGGCCACTTTATTAGTTACACCTGTCCAACTGTTTGTTAGCGCAATTTTCTAATCAGCCAATCACATGGCAGCAACTCAATGCATTTAGGCATGAAG 13540
   medaka --------------------------------------------------------------------------------AACAGAAGTT-------------ATGAAG 1806

 torafugu ATTTCC---TGAAAATCTGCT---------------------------CAAAAATGTTCTGTTATTTTGCTAAAAAGCCACCAAAACACCGG----TGGCCACAT 6318
zebrafish ACATGGTCAAGACAATCTGCTGCATTTCAAACCGAGCATCATAATGAGAAAGAAAGTCTGAGCTTTTC----AGAAACTGCTGATCTACTGGGATTTTCACACAC 13641
   medaka A--------AGCAA-----------------------------CAGAAATGT------------ATAAAAAA-----AAACCACTTT----TAAAGTCAT 1848
```
