# Supplementary material for: Evolution of the myosin heavy chain gene MYH14 and its intronic microRNA miR-499: muscle-specific miR-499 expression persists in the absence of the ancestral host gene
Source: BMC Evol Biol. 2013 Jul 6;13:142. doi: 10.1186/1471-2148-13-142 (PMC3716903; doi:10.1186/1471-2148-13-142)
Supplement: Additional file 3: Figure S3 — Intronic conserved regions in MYH14 among torafugu, zebrafish, and medaka. The red box shows highly conserved regions among the three fish species. Bold letters indicate 5′ and 3′ splice intron sites. Numbers on the right indicate the positions of the MYH14 (torafugu and zebrafish) start codon and mature miR-499 (medaka) 5′-end. Nucleotide sequences were aligned by CLUSTALW. [file 1471-2148-13-142-S3.zip › 1565208304857766_add3/1565208304857766_add3b.pdf]

```
  torafugu AAACTCCCCAGAAT--------------------------------------------------------------------ATATAATAGCA-----------C 6344
zebrafish AAACATCTCTGAGTTCACTGTACTCAAATGGCCTCCACAGTCACCAGATCTCACCATATTACCTTTGGGATGTGGTGGAATCGAAGATTCGCATCATGGATGAGC 13746
    medaka AAAAATCACAGAAA--------------------------------------------------------------------AAAAATTCTTA-----------C 1874

  torafugu ACACTATTAGACTGCA------------------------ATGATCCA----CTGTATGATAACTTTATAGTGCCACCTTT------------------------ 6397
zebrafish AGTCAACAAACCTGCAGCTGTGTGATGCTATCATGTCAATATGGACCAAAATCTCTGAGGAATATTTCCAGTACCATTTTGAATCTATGCCACTAAGGATTAAGG 13851
    medaka AGTCCTTT----TTCA------------------------TTTGACCA----TTTTCAGAGAATCTTACAGTG-------------------------------- 1915

  torafugu ---------------ATAGTGCCCTGGC------------------------TGCAGTGCCCCATTTG----AGGTTTTT------------------------- 6434
zebrafish CAGTTCTGAAGGCAAATAGGGGTCCAACCCTATACTAGTAAGTTGTACCTAATAAAGTGGCC-ATTGAGTATATGTCACTGTAACAATGAATGAAACGATAACTT 13955
    medaka ---------------AAAGAAATGCAGC------------------------TAAAGTGATTGATTCAAT--ATGTCATC------------------------- 1954

  torafugu -------------------CTACAGGTTTTGGTTTA-------------------------------------------------ATTAACTA------------ 6459
zebrafish CAGTGTTAAAAAACAACAACTATAACTTATTTTCTAAAAAAAAATAAAATAAAAAAAGAGGACTGGACAAGAGTCAAACCATCAAACCATCCATCGAAAAATA-- 14058
    medaka -------------------CCTCAGCTCCT-------------------------------------------------------ACCAACTACTGGAAAATAAA 1985

  torafugu --CATAGTCTGGGGA------------TTTTTGTGTAACTCTGAAG---GTGTCATTT------AAAATATATCTAAACATCATTTAATTAACTATTATGTTGGA 6541
zebrafish --TCTGGTTAAAGAAAAGTCATATTACGTTTTATAAAA----GAAGCCCAAACTATTCATTTT-TAAGCATGTTTAAGTATAAGTTGAGTTAAAAAAAGGTA-AA 14154
    medaka ACCATA--CAGAGAA------------TTTATCTACA-----GAGG---GGACTGTTCATGTCAAAAACATTTTCAGGCATGAAGTAGTCTAAGAGTAC-TACAA 2067

  torafugu AAACACTCACCCTTC----------------------------------------------------------ATTA----------------ATTCTTTAG 6569
zebrafish ACTTACTTATTTTGCCATTACAGTAGAAGAGCTACGTGTACAACCAAGATAATTATGAGACTTAATAAGACTAATTATACCTGTCTTTGTCA------TTAG 14251
    medaka GTATACTTAGTCCAC----------------------------------------------------------ACTAAAAAGTTTATTTTCATATACTATCT 2111
```
