# Supplementary material for: Evolution of the myosin heavy chain gene MYH14 and its intronic microRNA miR-499: muscle-specific miR-499 expression persists in the absence of the ancestral host gene
Source: BMC Evol Biol. 2013 Jul 6;13:142. doi: 10.1186/1471-2148-13-142 (PMC3716903; doi:10.1186/1471-2148-13-142)
Supplement: Additional file 4: Figure S4 — 5′-flanking conserved regions in MYH14 among torafugu, zebrafish, and medaka. The red and gray boxes show highly conserved regions between torafugu and medaka, and among the three fish species, respectively. Bold letters indicate 5′ and 3′ splice intron sites. Numbers on the right indicate the positions of the MYH14 (torafugu and zebrafish) start codon and mature miR-499 (medaka) 5′-end. Nucleotide sequences were aligned by CLUSTALW. [file 1471-2148-13-142-S4.zip › 1565208304857766_add4/1565208304857766_add4a.pdf]

(Sharmin et al., Supplementary Figure 4)

```
 torafugu GCAGCCTTGCTAACCCTTTCATTCTGCTTCACCTGA----CAGCAGGGGGCCACAGGGTGGCTCCCGCAGGGATCCCGGAGTGCGTCAGATTGTCTGCAG -3109
zebrafish -----------------------------------------AGCAGGA--------------TTGCGGTGAGATCCCAAAGTGCGACGGCTTGTCTGCAT -7348
   medaka GCGGCCTTGTTAACCCTTTCTGTCAGCTTCA-TTGAGGAGGAGGAGGGGGCTCCAAAGAGGCTGTCAGTGGGATGCTGGAGTGCGTCAGATTGTCTGC-- -5469

 torafugu GCCTGTGTTTGGATAAGGCGGCACGTCCATTGTCTCTGATGCCCCAGCTGGGTGGAGGTATGCCTCCGCGCTCCTGAAATAGAAAG-ACAGCTGAGAGCT -3010
zebrafish TG----GTTTAGATAAAGCCACGTGGC------------------------GCTGAGATAAACCAGCAT-------AAATAGAAAACCCAGCTAAAGACG -7283
   medaka ------GTTTGGATAAGGCAGTTTGCCCATTGTTTCTGATGCCCCAGCTGGGCCCAGGAATGCCTCCACCATCCCGAAATAGAAAG-ACAGCTGAGAGCT -5376

 torafugu GCCATGCATTCTCTGGGTAGGGCTGGTTGG-GCAGGCCGGGCCCACTGACCCAGACCACAACTTAGCACAAAGAGGGACGGGGTATTGTCTGGAGCCAGA -3109
zebrafish CCGGTA---TCTCAAACTGGGGCCAA--------------------------AGGCCACGACTTAAC-CAGTGAAAGACAGTCTTTTGTGTGAAGCCGCA -7213
   medaka TCCATGCATACTGAGGGCAGGGCTGGTCAGCGGGGCCCAGGTCCTCTGACCCACGCCATAACTAAGTACAATGAGAGATGGGCTATTGTCTGGAGC---- -5280

 torafugu GGTTAATAGCCGAGGTATTAAACTCCCCAACAATGGCTCACGAAGAGGCTGAT---TAACTCCG--CTGGGTTACTGAAGCCTGCGTGTGGGCGGGTGTC -2911
zebrafish GGGGCA--GCAGGACAGTCTCTCAATTCAACACTTGCT----AACAGACTAAT---TGACTTATACTTGTGTTGTC----TGTTTGTGTTGGCCCTGCTC -7126
   medaka --------GCAGAGGAATTACACT-------GATGGCT----AAAAGACTAATAAGTGGCTCTG--CTGTGCTGC--ATGTATGCGTGCGGGTAAATGAC -5203

 torafugu AGCGAGGTGCAGCC------GGCGTGTTCGGGTAATTGAGGCTTTAGTGTGCGTTTGTGCCACGCGTCAGCCCGTCTCCACTCTGACGAACCGACGGCTG -2722
zebrafish AG--------AGTATTGAATGAGATTATCAAAGAGCTGCGGTATTAACTGGTGTCTCCTCAATATGCTTTTCCGTCTCTCCACGGACGGATTGATGGCCT -7034
   medaka AG-GGGGAGTAGCAGAGCAGGGCGTGGAGGGGTAATTGGGGCTTTGGAGTGCAATTCTGCCACGCGCCTGCCTGACTCCACGCCGATGAACCCATGGCTA -5104

 torafugu AGAGCTGAGCCCACGCAGTTGCTGCAAGGTATTGTGGGCAGTCGTGTGATTACAG--------AAACATCCACTGTCCACCGATGCTTAGATCTGAAAGG -2630
zebrafish ATGACAGAGACAGTGCAGTTGCTGAAAGCTTTTGCAGAC--------GATCACAGCGATTATCAGACATCCA----------AGGTGTTGAACTGGATT- -6953
   medaka AGACCTGAGCCCACACAGTTGCTGCAAGGTATTGTGGGCA---AGGCGATTACAG--------AAATATCCACCAGCGATGAATGCTTTGATCCAGAGTG -5015

 torafugu TAACGAGGCTTCA-CGGGCCATTTAGGTC---------------------------------ACAGCCAGAGAACAAAAACATGG-------TCCGACAA -2570
zebrafish ----------------CTCCTGTAAAGGCCGTCGATGGGGCT---------------------------------------------------------- -6926
   medaka AATCACAGCTTCAGCGCTCAGGTTTAGGTCACTGCTGAAGCTCAGAGCAGGCAACTTCATAAACAGCCAAGAGTGAAGAAAACAGCTGCACATCTAAGAA -4915

 torafugu GCTGAAAATCTTAAGTTCTACAGGTTTCCTGCATCAGAACATGAGGAAGAATT--GGCCAAAG--------AAAATTCTGA------TCTTTTTTAGTTT -2486
zebrafish -------ATTTT--------------------ATTGAAACACTAAGA------------------------------------------TACTTTACTAT -6896
   medaka GACGAAAA-----------ACAAGTATC---GATCTAACCATAAACCATAATTTAAATTAAAGTACTTCTTAAAATTATAAATTCACACTTTTTTAGCCT -4829

 torafugu TT----TCTTTCTGCGTGTAAATCAGTT-----------GTTTTTGC---------------TTTTTCTAAAGATACAAATCCACTCTTTTAATTTTATA -2416
zebrafish TTTTCCTATTACCACTTGCAGGGCTGGGCG----------------------------------------------------------------TTTATA -6860
   medaka TTGATACATTTCTGCGTCTGACACTATTCAAGAGAAAGAGTTTTGGCAGAAAAAACTAAAATTCTTGCTAAAAA---AAAGCCAAAC----AGTTTCACA -4736

 torafugu GATATTTT-----------ACAGATATTTCAGATATTTACGATCTCGCCCTTTTCCTCTTCCAGAGGATCTTTTCCTCTGTCCTCTGAAAGTCCTGCTTC -2327
zebrafish TAAATTATCAGGTCTTACATGACAAATTAGGCATGTATGC-------------------------------------------------------GTTGC -6815
   medaka TTCATTGT-----------TTAAAAATTTGGAACGTTTGTTGTC----------------------GAT------------------AAAGTTTTACACC -4687

 torafugu AGGTTATCAGCAGGACCTCTGCAGCTGATGGAGGAATCTGACTGTCGTCTGCGATTAGACTCTACTGGGAATGGCTGCAATCATAATTCCAAATCTCTCT -2227
zebrafish AGACTGTCTCCC----------------TAAAGAGTTAGTGT----GTATGTG------------------TGGGTGTGTGTGTAAATTTCAGACTCTCT -6753
   medaka AGAC-----------------------ATGGAGAGTTGT-------GTGTATGGTGAAA-------GGCGATGGCTGCTGTCATAATTCCCCATCCCTCT -4624

 torafugu CCATCTCCCTGCTTAAGCCCGCTGGCATTCGGTGCATGATGATCTAAAACAACTGTGACCGACCCCCAGATCAAATCTTGATGCCC-------------- -2141
zebrafish CTGCTTCGGTGTCTAAGCCTGCTGGCATTTGGTGCACGATGATCTAAAACAACTGTGACCCAACTGCAATTGCAACTTTGATTCCAGGAATCCCTGTTTC -6653
   medaka CCATCTTCCTACTTAAGTCTGTTGGCATTCAGTGCACGATGATCTAAAGCAACTGTGACTGACCCCCAGATCAAGCATTCGTGCCC-------------- -4538

 torafugu ---------------------------------------------------------------------------------------------------- -2141
zebrafish AATCGGATGATCTGACTTAACTCTACTGTTTCGCAACAACTGATAAACATACACATGGTCCAAAGTCTGGCTCATTTAAAATGGTTTCCAGCTTGATTT -6553
   medaka ---------------------------------------------------------------------------------------------------- -4538

 torafugu ---------------------------------------------------------------------------------------------------- -2141
zebrafish TCTGTGGCTGTTTCACCGTTATTCTGGTCTTAAAAAAATAATTAGAAAATATTATTTAATTAAAAATTGTAAGTTTTAAAAGATTTTTCTTAGTAAATAT -6453
   medaka ---------------------------------------------------------------------------------------------------- -4538

 torafugu ---------------------------------------------------------------------------------GAAATGTCGCCTCCTCGCG -2122
zebrafish TTATTTTATTGTAACAAAATTGATATCAAAAATATTTTAGTTTTATTGCTTTTAGTTTTAATTTTAAACTAATGAAAATGGAAATATGTTCTTCTTACT -6353
   medaka ---------------------------------------------------------------------------------AAAATGTCAGCTTCTTGGT -4519

 torafugu CGCA--------------------------------CATCA-------------------------------------------CCGCGCGGTCGGCC-- -2099
zebrafish CCCAGGGCCATTTATATCGAAGTTGATATTTACCCGCATCATGTTGATTTTTCATAACATCTAATTTTTACGAGATGGTTTAGCCCAACCACCAACCTG -6253
   medaka TGTA--------------------------------CATCA-------------------------------------------CCAAACTGCCAGCA-- -4496

 torafugu ---------------------------------AGCCGATC----------------------------------------------------------- -2091
zebrafish GAGGACCAAGACATACACACATACGGACAATTTAGCTAACCCAATTTACCTATTGCGCATGTCTTTGAACTTGTGGGCGAAACCGGAGCATCTGAGGAAC -6153
   medaka ---------------------------------GACAAATC----------------------------------------------------------- -4488

 torafugu ------------------GCTGCGAAGGCGCTG------------------------------------------------------------------- -2076
zebrafish TGATGGTTGATTCACTGACCCACTGAGCCACTGTGGCACCCACATATATTGTTATAACTGTTTTTTTTTTGTTTGTTATAATTCACCACTGCTATTTTAA -6053
   medaka ------------------CCCAGCAGAAC----------------------------------------------------------------------- -4477

 torafugu ----------------------------------------------------AGGACTGT------------------------------GCTCCAGTGT -2058
zebrafish AAATAAATAACCAAAATATAATTTTAAAATCGACAATTTTACAATTAGTTCTAAAATAATCAAATCAAAGTCTGCTGATGTAAATTAAAAAATTTATCCA -5953
   medaka ----------------------------------------------------AGAACAATCAGATCAGCC--------------------GCTCCTTCAA -4449

 torafugu TTTCGGGACT----------------CCAGCTGGT----------------TGTCGGTTTGGTCATTTTATCTGGGAGGGTC------------------ -2008
zebrafish TTTCAGCAATGTTAATAATAAGAGCCTTTATTAATGATTGAGTGCCATTAATGTATCATTGAGCACTACAACTATGAGAATAACTTCTGAAGAATCAATT -5853
   medaka TTTC----------------------TTTTTTAGT----------------TGTTTCTTT-----TTTTATAT--------------------------- -4419

 torafugu ------------------------------GTTTCAAGCGGCGACAAA---------TGTCTGATAATCA-------------------------AAACA -1972
zebrafish GACACTTGATTGATGATGCTGAAAATCCTTATTTGAATAATAGAAATAAAATACATGTAAAGTATATTCATATAGTTTCTACTGTATATTTGATCAAACA -5753
   medaka ------------------------------ATATAAAAAGGGGAAAAAAAGAATTTGCACTTTAGTTTCA-------------------------AAACA -4374

 torafugu CCGATGGGCTTGGCTGCATTCAGGACTAAATGATTTTGAGGAGCACAGGGGAC----------------------------------------------- -1919
zebrafish CATGCATCTTATGCTGTAAAAAGTAGTTGCTGTCTTAAATGAACCCAGCAGACCCAACTACACAACGTCATGAGAGGTTAATATTGTCCAATGAAGACGT -5653
   medaka TGAGTAG---TAGCCACATCAGGCTCTAAATGATTTTGAGGAACACA----------------------------------------------------- -4330

 torafugu -------------------------------------------GTAAAGAGAGCTG-------------------------------------------- -1906
zebrafish AAAATGATGTTGATATTTGGTTAATTTTAGGTTGTGTTGGAAAGTGAGGAAAATCAAACATCAGGCCGGCATCTTAAACCAACGCCATATTGATGTCAAA -5553
   medaka -------------------------------------------GCAAGGAGAGCC--------------------------------------------- -4318

 torafugu ---------CTAGAAATCAGCTGTTGC---------------ACTGATCTGCAGTGTAGCAGCGCAGA-------------------------------- -1862
zebrafish TACTGACATTTATACATCAGGTATGGCAACCAAAATCCAAGGTCTGATAGACGTCAAATTGGTAAACACAACCTCAAGCTGTAACATCATTAGACGTTGA -5453
   medaka ---------TGAGAAGTCAGCTGTTGC---------------TCGGGC--------------TGCAGA-------------------------------- -4288

 torafugu ------------------------------------GAGATGTGCAGGG-----------GGACAACACTCACTGTGTGT-------------------- -1829
zebrafish TATTTGGTTGTTTTTAGGTTGTGTTGGAAAATAACCAAAATGCAACGTATGTCCGACGTTGGACAT---TGACGTTGGGTTCTGACGTCTGACGTTTTCA -5356
   medaka ------------------------------------GAAATGTGCCGGGACGCCGGCTTTGCACGTCCCGGACTGTGGGGTCTGTCA------------- -4237

 torafugu ---------------------------------GTGTG----------------------------TGTGTGTGTGTTTGGTGCAGGAGTATGCGGACT -1790
zebrafish TTTCCAAACAAATTGAAATATTCGAAGACGTTGGGGTACATCGTCAATCTGACATCATATTGACATCATGTGTCTGCTAGGAAGTTGAATCAAATTAACT -5256
   medaka ---------------------------------GGGTG----------------------------TGTGTGTGTGTGTTTGAGCCGCAGTTTGTGAACT -4198

 torafugu T------------------------------------------------------------ATTTAAAG------------------------------ -1782
zebrafish TTACAAGTCATTTCAATTTATATCAGTTAAACTGACTTAAAACATCAAGTACCAATCGAAAAACTTAAAAAAAATTAAGTCAAAACCTTTTCTTTTTTTT -5156
   medaka T------------------------------------------------------------ATTTAAAG------------------------------ -4190

 torafugu ---------------------------------------------------------------------------------CC-AGCA------------ -1776
zebrafish TTAAGTATCACAGAAAAATACGTTGTAAAGGTTTTTCATAACATCATTTTTCCAGTGTAGACAACAAAGAAGATTTTTTCCAAACATATTAACAAATC -5056
   medaka ---------------------------------------------------------------------------------CCGAGCA------------ -4183
```
