# Supplementary material for: Evolution of the myosin heavy chain gene MYH14 and its intronic microRNA miR-499: muscle-specific miR-499 expression persists in the absence of the ancestral host gene
Source: BMC Evol Biol. 2013 Jul 6;13:142. doi: 10.1186/1471-2148-13-142 (PMC3716903; doi:10.1186/1471-2148-13-142)
Supplement: Additional file 4: Figure S4 — 5′-flanking conserved regions in MYH14 among torafugu, zebrafish, and medaka. The red and gray boxes show highly conserved regions between torafugu and medaka, and among the three fish species, respectively. Bold letters indicate 5′ and 3′ splice intron sites. Numbers on the right indicate the positions of the MYH14 (torafugu and zebrafish) start codon and mature miR-499 (medaka) 5′-end. Nucleotide sequences were aligned by CLUSTALW. [file 1471-2148-13-142-S4.zip › 1565208304857766_add4/1565208304857766_add4b.pdf]

```
 torafugu ---GCTATGGGGACTTT------------------------------------------------GGGATTCATCTCCTCCTCATGTGTTATCAGCATGC -1726
zebrafish ATTGCATTACGAACTTTTAACCAGTGCATAATAAAAAATAAAATAAATAAATATAAAATCATCATCAAATTTAATTATCCCTCACGTTTTATCAGTCTGT -4956
   medaka ---GCTCTGGGGACTTTC-----------------------------------------------GGGATTCATCTCCTCCTCATGTGTTATCAGCATTC -4132

 torafugu CAGCTTT------TCAAATGGAGCAGCATGTGAGACAGTACACAAAACA------GCCTCTCTGAAGAGCCCCTCGTCCCAGAATAGCTCCGGCCCGTTC -1638
zebrafish TAACGTTTTTTTTTTAACACGACTAGCAAGTGAGGCTGTACATAACCCAGCTGCCACACTCATGAAG-------CGTCCCAGAATAGCTCTCAACT---- -4867
   medaka CAGCTTT------TCAAATAGTGCACCAAGTGACATGGGACATAAACCAGCCTGTGCCTCTGTGAAGTGACTCTTGTCCCAGAATAGCCC---------- -4047

 torafugu AGGCCCGCTCTATCTCGCTGATGTCCAAGAAAGTTGGAGCCGTCG-GGCAACACCTCAATCTGCCACAGCAACAGATA-----AGCGGCATCAACAAAGA -3968
zebrafish -GGCTCACTTTATCTGACCCAAGCGCAACAAACCTTCAGTCGTCAACGTAACACTTCA---CGGTTCGCCAACAGATAGCAGCAGCTATATCGACAACAA -4771
   medaka -GGCCCGCTCTATCAGACT---------GAAAGTTTCAGCCATCACAGCGGCACC----TCTGCGCTCGCCACAGATA-----A-CGCTGTCAACAAAGA -3968

 torafugu TGGCCACCCGCCGCGT---------------------------------------------------AAAAACTTCC----------------------- -1544
zebrafish TAATTGGTACTTATTCCACCACAATACCACTTGATTTACAGCAGCAACAGGTTTCACAGCGGCCGGTAAAATCTACCCACTGACTTCGTTCTCAAACAAT -4668
   medaka TGGCTGACTCTCAAGTCA-------------------------------------------------AAAAACATCC----------------------- -3940

 torafugu -----------------------ACACGGGTTAT---------------------------------------------------------------TT- -1505
zebrafish AGGCCTGGTCTGTTGCTGTTGGTCTGCCAGGAATGCCAGTCTTTGGCATGAGGCGCGGATGGCGTATTCTTGTCTATATCAGGACACTATGAGCCTCCTC -4571
   medaka -----------------------CTGATAGGAAT---------------------------------------------------------------TTG -3926

 torafugu TCCCCTTTAGGCGCTCCGTCGTACTCC-------GAGCACCCGAGAGTCACTGCCTCATCCGTGTGG--------------------------------- -1445
zebrafish CTCCCCTTTTGCTCTTTACCCCTTTTCCACATACAAGGACACTGAGCTCAAGGTCAGATCCGCTGGGGCCGCTATGTAAAAAGAGGCGCTCAATTACTGC -4471
   medaka TTTCCTTGATG-----------------------AAGCGAAGAGAAAGCCCTTTCTGGAGCGTTTGG--------------------------------- -3882

 torafugu ------------------------CT-----------------TTCGCAGATTCCAGCGCCGCT------------------------------------ -1422
zebrafish CTGCATAATAAGCTCAATTTAGGCCTGTTATTTTAACACTTTGTCTGGAAAGTTCTGCGCCACACCTGCCATTTTGAATATCAAAATTGTGTGTTGGGAG -4371
   medaka ----------------AACCAGGCCT-----------------TTTGCTAAATTTGGCAC---------------------------------------- -3855

 torafugu ---------------------------------------------------------------------------------------------------- -1422
zebrafish CGAAGTGGGCTGGATGTGTGAATTAATTATTATATATGAGTAGATCTGAAAGCACATGTTGTCAGTACTGTAAGAACATGTTTAGGAGTCACATAATGAT -4271
   medaka ---------------------------------------------------------------------------------------------------- -3885

 torafugu -------------------------------------------------------AACAG-CTAACAGCCAATCTC-----------------CCTATCA -1395
zebrafish TAAACAAACTGCCTGATTCATTTAATCACAGTAAATTGTGTTTTTCAGAAATAAAAACATTTTAGTGTCCCTTATCATGTCAAGCACAAGGGACCCATTA -4168
   medaka -------------------------------------------------------AACGGTTTAACGCTCAATTTC-----------------CCCATCA -3827

 torafugu GGAATGC-------------------------------------------TGGACTCTGGCTTTGTCTATTCTGAG------------------------ -1362
zebrafish GCAAAGTATTTAAAAAAATAATTCAACTAAAAACTATCAAGTAAAATAAGATGAATATGTTTTTTTCTTTTTTGAGGTAATGTTTTTGATAGAAAAACAA -4071
   medaka GTAATA--------------------------------------------------CTGGCTTTGTCTATTCTGAG------------------------ -3801

 torafugu ---------------------------------------------------------------------AAACTGTGGAC-------------------- -1351
zebrafish AACATTAACTATTTTTGTTAACTTTATTTTTGTGGATTTAGTTTTAGTTAACTGTAAGATATTTGTTTTAAAATGAGGTTTATTTGCAATCAAATGCACA -3971
   medaka ---------------------------------------------------------------------AAACTGTGGTCCACTCT-------------- -3784

 torafugu ---------------------------------------------------------------------------------------------------- -1351
zebrafish AATACATATATTTATAAATGTATATAAATATTTCTACATGAACATAATATGTGTACAAACAATTCATATATAACTGCAAATAATTATAAATAAAAGTATA -3871
   medaka ---------------------------------------------------------------------------------------------------- -3784

 torafugu ---------------------------------------------------------------------------------------------------- -1351
zebrafish ATTATATTTTTACATTTTTAATTATCTATATACATTTTTTTGACATTTTGAAAATAATCAGTTTTGAGTCATTTTTGTATATTGTTAATAAATAGTTCCA -3771
   medaka ---------------------------------------------------------------------------------------------------- -3784

 torafugu ---------------------------------------------------------------------------------------------------- -1351
zebrafish TTAGTTTTGTTTTGTAATTTTTTGTAGTAGAAATTTTGTTATTGTCATTTATATTAGCTTTAGTTTAGTTATAGCTATATAGTATTTACTAATGATGGTA -3671
   medaka ---------------------------------------------------------------------------------------------------- -3784

 torafugu -------------------------------------------------------CCCTTCCCCA----------------------------------- -1341
zebrafish TTTTTACCTTTGTTTTAGTTCAACTAAATGACCCCTGAATTATTAGCCTGTATATTTTTTCCTCAATTTCTGTTTAACGGAGAGAAGATTTTTTTAAACA -3571
   medaka -------------------------------------------------------CCCTCCCCCACTT-------------------------------- -3771

 torafugu ------------------------------------------------------CCCTGTGCCGCACTGTCACC-------------------------- -1321
zebrafish CATTTCTAAACAAATAAACTTTTTATAAGTCAATTCTAATAACTGATTTGTTTTTTCTTTGCCATGATGACAGTAAATAATATTTGACTAGATTTTTTCA -3471
   medaka ------------------------------------------------------CCCCGAGCAGCACTGTCACC-------------------------- -3751

 torafugu ---------------------------------------------------------------------------------------------------- -1321
zebrafish AGACACTAGTATTTAGCTTAAAGTGACATTTAAAGGCTTTACTAGGTTAATTAGGTCAACTAGCAGGTTAGGGTAATTAGGCCAGTTATTGTATAGCGAT -3371
   medaka ---------------------------------------------------------------------------------------------------- -3751

 torafugu -----------------------------------------------------------------------------------CTGCTCTCATACCAGGC -1304
zebrafish AGTTTGTTTTCTAGACAACCAAAAAACATTTATAGCTTAAAATAATTTGGACCTAAAAAAGATTTTCAAAAAAATAAAAAAAACTGCTTTTATTCTAGCC -3271
   medaka -----------------------------------------------------------------------------------CTGCTCCCATACCAGCC -3734

 torafugu ACCA-------------ACCTCTCCCCTT---------------------------------------GCTCTG-------------------------- -1282
zebrafish AAAATAAAACAAATAAGACTTTTTTCCAGAAGAAAAAATATTATCGGAAAGACTGTGAAAATTTCCTTGCTCTGTTAAACATCTTTGGGCTTAGTGCCTT -3171
   medaka ACCA-------------ACCTCTCCCATGA--------------------------------------GCTCTG-------------------------- -3711

 torafugu ---------AAGGTC------------------------------------------------------------------------------------- -1276
zebrafish TATTAATCTGGGGTCGCCACAGTGGAATGAACCACCTACTTATCCATCATATGTTTTACACAGCGGATGCCCTTCCAGCTGCAACCCATCACTGGGAAAC -3071
   medaka ---------AAGGTC------------------------------------------------------------------------------------- -3705

 torafugu ---------------------------------------------------------------------------AGATGTGGTGAGGTCAGTCAGGCAC -1251
zebrafish ATTCATACACATTCATTCACACACATACACTATGGAATATTTAGCTTATTCAATTCACATATACCACATGTTTTTAGACTTGTGGGGGAAACCGGAGCAC -2971
   medaka ---------------------------------------------------------------------------AGACTGGCTGGGG-----CAGGAGC -3685

 torafugu CCGAA--------------------------------------------------------------C--------------GCAA-------------- -1241
zebrafish CTGGAGGATACCCATGCCAACACGGGGAGAACATGCAAACTCCACACAGAAATGCCAACTGACCAAGCCATGGCTCGAACCAGCAACCTTCTTGCTGTGA -2871
   medaka GTGGA--------------------------------------------------------------CCGTGA---------GCAG-------------- -3670

 torafugu ---------------CACTTCATCA--------------------------------------------------------------------------- -1231
zebrafish GGTGATTGTGCTATCCACTTCGCCACCGTGCTGCCTATTTATATTTTATATATAAAAATATTTTTATTGTCTTAACTTTTAATTTAAATAAATACTTGCA -2771
   medaka ---------------CGC---------------------------------------------------------------------------------- -3667

 torafugu --------------------------------------------AGAAATAGC----------------------------------------------- -1222
zebrafish TAAGTAGTGCATGCATACTTTTAAATACATTTTTTCAAGAAAACATAAATAGCAAGAGAAGTTAATTTTATTGTTTTACTTTTTTGTTTTTAGTGTTTCA -2671
   medaka --------------------------------------------AGAGCTCGCGAGA------------------------------------------- -3654

 torafugu ---------------------------------------------------------------------------------------------------- -1222
zebrafish TTTTATTAAGTTTACATTTTAGTATAAACCAAAATAAATATAAAAGAATAAAAAAATATAAAATAAATATTTCCATAGCTTTTTATTTTTTCTCCTGTTA -2571
   medaka ---------------------------------------------------------------------------------------------------- -3654

 torafugu -------------------------------------------------------------------CAATGTCCA------------------------ -1213
zebrafish TCATTATGTTTCAGAAATGATGTTATCAGCTTTAGTTTAGGTTAACTATAACATCTGCTAAGTGACTCAATGTAAATATAAATAATAACTTATAGAATTA -2471
   medaka -------------------------------------------------------------------CAATG---------------------------- -3649

 torafugu -----------------------------------------------------------------------------GAAAGACGTCTCCCGTGTGCA-- -1192
zebrafish TATAAAATAATTATAACTGTCATATCACGTAAAGACAAATTATCAATTTGAATTGTCACTCACTCACTCACTCAATCAGAAAACTGCACCGGTATCCATG -2371
   medaka -----------------------------------------------------------------------------AGAGGACGG-TCCAACATGCA-- -3629

 torafugu ---------------------------------------------------------------------------------------------------- -1192
zebrafish AAGGAAAGTATCTATGCAGGAAACAACATCCATATCAATTATACTGCTATTATCATTAGGGCCAGTGCATCATGGCTTATATTGGCATGTATTTTTAGCG -2271
   medaka ---------------------------------------------------------------------------------------------------- -3629

 torafugu ------------------------------------------------GGAACACAGACCGCGTCCTGACA----------------------------- -1169
zebrafish TCCCATATCATGTCGAGCACAAGGGACCCTTTAGCAATTTGGTCTAGGGTTGCATTTACTGCGGCCACACACACACACACACACACACACACACACACAC -2171
   medaka ------------------------------------------------GTGGCAT---CCGAGTTCATCCA----------------------------- -3609
```
