# Supplementary material for: Evolution of the myosin heavy chain gene MYH14 and its intronic microRNA miR-499: muscle-specific miR-499 expression persists in the absence of the ancestral host gene
Source: BMC Evol Biol. 2013 Jul 6;13:142. doi: 10.1186/1471-2148-13-142 (PMC3716903; doi:10.1186/1471-2148-13-142)
Supplement: Additional file 4: Figure S4 — 5′-flanking conserved regions in MYH14 among torafugu, zebrafish, and medaka. The red and gray boxes show highly conserved regions between torafugu and medaka, and among the three fish species, respectively. Bold letters indicate 5′ and 3′ splice intron sites. Numbers on the right indicate the positions of the MYH14 (torafugu and zebrafish) start codon and mature miR-499 (medaka) 5′-end. Nucleotide sequences were aligned by CLUSTALW. [file 1471-2148-13-142-S4.zip › 1565208304857766_add4/1565208304857766_add4c.pdf]

```
  torafugu ---------------------------------------------------------------------------------------------------- -1169
zebrafish ACACACACACACACACACACACACACACACACACACACACACACGTGCACACACACGCACACACACGCACAATGACTGCCTGTGCTGGCGTTTACAACAA -2071
    medaka ---------------------------------------------------------------------------------------------------- -3609

  torafugu ----------------------GTAGCGTGACTAAAGAGACAGC------CCATCATTT----------------------------------------- -1138
zebrafish AGGATTCCGCAAACAAGCAATTAGAGTAAGAGAGAAGAGCGAACACATGGCCATTATGTTCACCTAATACTTGTCCTGTATGTTGAGGAGAGAATAGTTG -1971
    medaka --------------------------------------------------CCATCATCT----------------------------------------- -3600

  torafugu --------------------------------------------------------------------------------------------------   -1138
zebrafish ACGAGTTATTGAGGAGATGTTAAAGAGCCGGTGGAAAGGGTGTTGCTTGTTGCCGCTGAGTGGATCTGTAACCTTTATCCAGGCCGGGACCATGGGGGAG -1871
    medaka --------------------------------------------------------------------------------------------------   -3600

  torafugu GGTTT--AGGGCCGAGGCTGCT-----GGTG-----------------------------------------------CAACACCT-------------- -1106
zebrafish TTTCAGAGACCACCGCCGCTGCTCAGATGACTGTCGATGTTGAAGACAAAATAAAACTCTGAGAAAAACAAACAAAAAACATGTTATTAAACAAAAATAA -1771
    medaka GGCTCTAAAGACCAAGA--GCTTCAAAGGAG-----------------------------------------------AAACACGC-------------- -3563

  torafugu ------------------------------------------------TTGCTGCCTGCAA--CTTCACAG----------------------------- -1085
zebrafish ATGATAATAATAATTAAGATTTAAGAAATATGTTGATTAATAATTATTATTGCCTTAAAAACATTAAAGTGTTAATGCACATTACATTTATTCTAATATG -1671
    medaka ------------------------------------------------TCGTGTT--------CTTCAAAG----------------------------- -3548

  torafugu ---------------AATCTGTAACCGCTC----CTTAGACTCG-------------------------------------------------------- -1060
zebrafish CAATACAAATGCAACTTTTTGGTTCCCCAGTTTTGAAGTTTGATGCAAACTGAATAAAATTTTTTTTAAAAATCAAAGACATAAATTTTAAAGTGTGAT -1571
    medaka ---------------GCTGCTTGGTTTTTC----TTAAACCTTG-------------------------------------------------------- -3523

  torafugu -------------------TAAAA------ACTGTCCCTCTCTTTTCACCGAAATCAGGCT------------TTTTATCGCA----------------- -1014
zebrafish TTCCCTCATAACTACACTAAAAATCCTGGTTGCCTTAAATTTTTAAGCTGAATCAAATTAACCTTATATGAGTCCATTGAATTTATATGATGTTAAACTG -1471
    medaka -------------------GGAGACGCTGGACTGT---------------GAAGCCACACT------------GCCT----------------------- -3492

  torafugu -----TCGAGAGCCGCGC---------------------------------------------------------------------------------- -1001
zebrafish AATTTAAAAACCTTGCATAACTTATAAAGTTAAGTTAGAACATGCTTGAATTAGTTTAGTAAAACATATGTTGTCAGGACTAATAGATCATAAAATTTTT -1371
    medaka -------------CTTGC----------------------------------------------------------------------------------- -3487

  torafugu --------------------------------------------------CATGTT-------------------------------------------- -995
zebrafish ACAGTATGCTAAAGTAAGAAAAAAAAACTTTACATGATTAATTTATTTCATGTTACAAAAAACTTTTTAAAGTGAGAATGAAACTTGTAAAAGCAAAAAA -1271
    medaka --------------------------------------------------CATCTT-------------------------------------------- -3481

  torafugu -----------------------------------TATCTCTATTAATATAA-------------------------TGATGTGTATTT-----ATAGTA -960
zebrafish AAACATGAAGTTAATACAACAACAGTAACAACACATCTTTAAAAAATATTAATATTCATGATAATATTAATACATTTGATGCAAATTTGACTAATAGCATA -1171
    medaka -----------------------------------CCTCTCTAAAAATAAAA-------------------------TGATGTCTATTT-----ATAGTG -3446

  torafugu ----------GCCTGTTTAATGCCGTGCCGCTCCTGAAA----------TAAGCTCTTCTAAGGTTGCATCCTCTTTAGC-------------------- -900
zebrafish ATTAATATGTATGTTTTATATTGCTGCATTCCTGTTATTGTTGTTTTAATTTCTTTTTAGTTT------TTTTTAGTTAACTAATAAAAATAAAAAAA -1077
    medaka ----------GCAAGTTTAATGCCACACAGATCCTGAAA----------TAATCTCTTCTAAAGTTGCCTCATCTGTGGTAG------------------ -3384

  torafugu --------------------------------------------CACACAATAGCAAACATCTCTT---------------------------------- -878
zebrafish GTATTTCCCTGAAAACTATCAAGTAAACTATGATGAATACTAAAAGAAAAAGAAAACATTTTTTGGATTTTGTTTTAGTTACTGTAGGTTACTTGAAAAT -977
    medaka --------------------------------------------CACACAATAGCAAACATC-------------------------------------- -3366

  torafugu -----------------------------------TTGGCACTATTGCAGGAC---GATTACCT-AAGGGCAACATGA-------GTTCATA-----AGC -829
zebrafish GAGAATGAAAATAAAAGCCATAAACATGAAAAAATGGTCTTATTGCTGTTTTCAATTAGTTTTAAAGAAAAGCTGAAAATGAGATGCATTTTCTTAGCAA -877
    medaka -----------------------------------CTGGC-----TGCAGCCT---GTTAGGCTGGAGGGCAACCTGA-------GTGCAT------GGC -3322

  torafugu AGCA-----------------------------------------------GTCATTTACAGAGAGGGGGGCTTCACCTCTATTTATCAGA--------- -785
zebrafish TAGCTAAAGTAAAACAGGTTTAATGGGTTTATTTATTATGGAATTTATTTTCATTTTAA-------------CAAATCTTCTGATCAAAAACTAAAATTG -790
    medaka ATCAAT---------------------------------------------GTCATTCTGAGA---------TTCTCAGTCTGTGATCAGG--------- -3285

  torafugu -------AGGGTGACGGTGGAA------------------TCTA-------------TAAAACAACTATACTATAGATGGGTATGTCTCCAA--ATAAAT -725
zebrafish ACTTTAGTTATATGGTTTTAAGCTAATAATTACCCTGTTCTGAAGTGTGAATAACTAAAATAAATTAAATAAAAATATACTTGTATTTAAGTTTCATCTG -690
    medaka -------AGCTCTGTGGTTGAAAGAGATGATTAGACTCCTTCTA-------------TGAAA---------TCTAAATATATATTTGACCCC--TTTCTT -3216

  torafugu GGGTTTCTG--------------GCAGTTTTCTTCTGTTTG--------CTGAC--------------------ACAACTCACCACTTGATGAGTCCTTC -667
zebrafish TATTTTGCACTGCACCATATTATAATTTCCTTTTTTATGGAAAAG---------------------------------------TTGACATATTTAACTT -629
    medaka TAATTTCTGCTCT----------AATATTTTATTTTCCTTGTATCAATCCTAACTTTCAGATTACGTCTTAGCTACAACTCGTGACTTAA--AGTTCTGT -3128

  torafugu AAAATGCACTGCGAAAAAACTA---AATCTGCCCATGCCGG------GATGCTCCTGAT-GCAATCCTCTCCAG--------------------TGCAT- -598
zebrafish AGAAAATTCCCAGTATAATTTCCAATATCCCCATGCAAGTTTAATAAAGTTGAAAATGGCAAGCAGTTTCGAATCATATTCA----------TGCATCCT -539
    medaka CTATCAAGTTTCAAAGAAGTAAGGAATTCCTCCACGCTGG------GATGCTCCCAGT-ACAAGGCTCTTCCACTCCCTGACAGTGTGAACGCTGCAT- -3036

  torafugu ------AAAATCACTGAGATACAAAAATAGCC-GGCTTGGGTTCAGCGCAATGACAAAAACGCAGCTT-TCCTTTTGTGTCAATTGAGACAGGCGCTCCG -506
zebrafish TTTCAATATAATGCTGCTATAAAAATAACTAAGCTTGGGTTTGG-GTAAAGATGAAAATATGGCTTGCCCTTTTGTGTCAGTTTGGGCACATGGTGAGCA -440
    medaka ------GAAATAAGTGGGATACAAAAATAGTC-TGCTGGAGTTTAGCGCTATGACAAAAACGCTGCTT-TCCTTTTGTGTCAATTGAGACAGGCGCTCAG -2944

  torafugu CA-CCAATGGGCGCCGGAGGTGGTGGCATATGCTAATCAGTGACAAATGTCCACTGACCGTGACCTTGAGAAACACGGTATAAAGGAAGACCCCATCTCC -407
zebrafish -CCAATAGGCATTGGAGGAGGCGACACACGATAAT--TTCTGACCATGACCTTGG--AGGAGATGATA--AAAGGAGGT-TA--GCCACT------CCAG -356
    medaka CAGCCAATGGGCTTCTGAGGAGGTGGCACATGCTAATGAGGGACAAATGTCCACTGACCGTGACCTTGGGGAACGCGCTATAAATG-TGTGCACACCTCG -2845

  torafugu ACAGGCTGTTATTTCGTTCTGATCGCCCCGGGGTCA -371
zebrafish -------------CCATTCAGTCCCTGTTAAGGTCA -333
    medaka GAGGGGCG-CATTTCGTTGTGCTTTCTCTCAGATCA -2810
```
